# Health systems constraints and facilitators of human papillomavirus immunization programmes in sub-Saharan Africa: a systematic review

**DOI:** 10.1093/heapol/czaa017

**Published:** 2020-05-03

**Authors:** Edina Amponsah-Dacosta, Benjamin M Kagina, Jill Olivier

**Affiliations:** c1 Vaccines for Africa Initiative, School of Public Health and Family Medicine, Faculty of Health Sciences, University of Cape Town, Anzio Road, Observatory, Cape Town 7925, South Africa; c2 Health Policy and Systems Division, School of Public Health and Family Medicine, Faculty of Health Sciences, University of Cape Town, Anzio Road, Observatory, Cape Town 7925, South Africa

**Keywords:** Africa, cervical cancer, health systems, human papillomavirus, HPV vaccine, immunization, national immunization programmes

## Abstract

Given the vast investments made in national immunization programmes (NIPs) and the significance of NIPs to public health, it is important to understand what influences the optimal performance of NIPs. It has been established that well-performing NIPs require enabling health systems. However, systematic evidence on how the performance of health systems impacts on NIPs is lacking, especially from sub-Saharan Africa. We conducted a qualitative systematic review to synthesize the available evidence on health systems constraints and facilitators of NIPs in sub-Saharan Africa, using human papillomavirus immunization programmes as a proxy. Fifty-four articles published between 2008 and 2018 were found to be eligible. Data extraction was guided by an analytical model on the interface between NIPs and health systems. A cross-cutting thematic analysis of the extracted data was performed. This systematic review provides evidence necessary for informing ongoing health systems strengthening initiatives in sub-Saharan Africa. There is evidence to suggest that NIPs in sub-Saharan Africa have surmounted significant health systems constraints and have achieved notable public health success. This success can be attributed to strong political endorsement for vaccines, clear governance structures and effective collaboration with global partners. Despite this, significant health systems constraints persist in service delivery, vaccine communication, community engagement, the capacity of the health workforce and sustainable financing. These constraints could derail further progress if not addressed through health systems strengthening efforts. There is a need to expand the research agenda to include the comprehensive evaluation of health systems constraints and facilitators of NIPs within sub-Saharan Africa.

**Key Messages**

- National immunization programmes (NIPs) are embedded within health systems. However, the interactions between NIPs and health systems are poorly understood.
- This systematic review provides the evidence of how NIPs and health systems interact by reporting on the health systems constraints and facilitators of NIPs in sub-Saharan Africa.
- Strong political will, clear governance structures and effective collaboration with global partners have been major facilitators of NIPs in sub-Saharan Africa. Despite this, significant health systems constraints persist in service delivery, vaccine communication, community engagement, the capacity of the health workforce and sustainable financing.
- The findings of this review have relevance for ongoing health systems strengthening initiatives in sub-Saharan Africa, especially where NIPs are concerned. By providing a better understanding of what works—and does not work—for NIPs, health systems strengthening initiatives could be better designed to adequately respond to the burden of vaccine-preventable diseases in sub-Saharan Africa.

## Introduction

It has become increasingly apparent that some of the major challenges experienced in scaling up the performance of national immunization programmes (NIPs) in low- and middle- income countries (LMICs) are not necessarily programme specific, but rather challenges in wider health systems functioning (SAGE, 2016). This is in line with the notion that NIPs exist in a continuous interaction with the health systems that deliver them (World Health Organization Maximizing Positive Synergies Collaborative Group, 2009; Shen *et al.*, 2014; SAGE, 2016). As such, major health systems constraints could have a significant impact on how NIPs perform. For example, financial, technical, logistical, political and socioeconomic constraints have been cited as negatively impacting on the overall performance of NIPs in LMICs (SAGE, 2016). Unfortunately, these health systems constraints appear to be even more prevalent in sub-Saharan Africa (SAGE, 2016).

In sub-Saharan Africa, NIPs have undergone steady advancements since the establishment of the Expanded Program on Immunization in 1974 (WHO, 2013a). Tremendous progress has been made in increasing access to lifesaving vaccines and reducing the burden of vaccine-preventable diseases in the region (Arevshatian *et al.*, 2007; Machingaidze *et al.*, 2013). Despite this, sub-Saharan Africa continues to lag in meeting global immunization targets (SAGE, 2016; Mihigo *et al.*, 2017). Although substantial investments have been dedicated to NIPs in sub-Saharan Africa, this has not been enough to improve their performance to levels required to significantly reduce the disproportionate burden of vaccine-preventable diseases in the region. In this regard, global actors have recognized the harrowing state of health systems within sub-Saharan Africa as a major barrier to achieving further improvements in the performance of NIPs (SAGE, 2016). This has contributed to making health systems strengthening in sub-Saharan Africa a matter of global public health priority (SAGE, 2016). It has been argued, however, that while ongoing health systems strengthening efforts are absolutely vital, they have not been able to achieve their intended outcomes (Marchal *et al.*, 2009; Chee *et al.*, 2013). Some of the challenges faced have been attributed to the fact that health systems constraints are not adequately defined and as such interventions are often poorly designed and weakly targeted in the long term (Marchal *et al.*, 2009; Goeman *et al.*, 2010; Chee *et al.*, 2013). Systematic evidence on how health systems constraints (and facilitators) impact on the performance of NIPs in sub-Saharan African could be very useful in better informing health systems strengthening efforts in the region. Unfortunately, such evidence is lacking.

**Figure 1:**
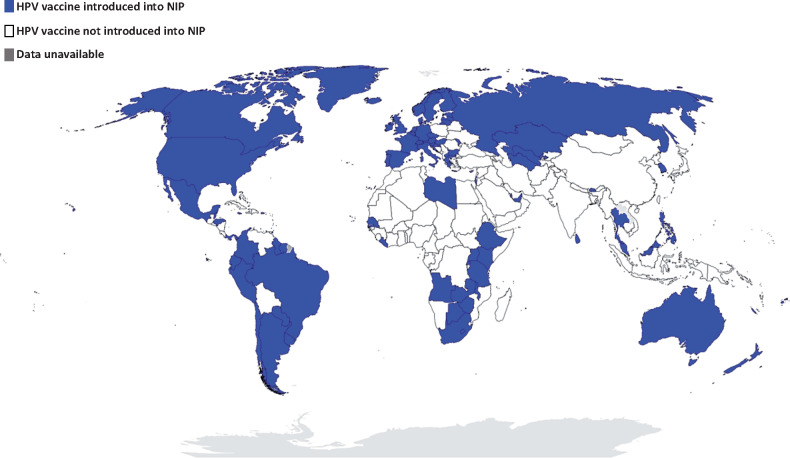
Global progress in the implementation of nationwide HPV immunization programmes. Data from countries with planned or partial HPV vaccine introduction, or HPV vaccine demonstration projects, are not represented here [drawing on data from http://www.hpvcentre.net, WHO (2019), Gallagher *et al.* (2018), LaMontagne *et al.* (2017) and Herrero *et al.* (2015)].

We report on a qualitative systematic review study that sought to determine how health systems constraints and facilitators impact on the performance of NIPs in sub-Saharan Africa. The NIPs serve as a platform for the delivery of several immunization services. These include routine childhood, adolescent and maternal immunization programmes, mass immunization campaigns, outbreak response or emergency vaccination services and introduction of new, improved or underused vaccines into existing NIPs. Ultimately, an in-depth review incorporating the mass of these services would not be appropriate. As such, a single immunization programme—the human papillomavirus (HPV) immunization programme—was selected as a proxy or tracer immunization programme for the purpose of this systematic review. Our interest in HPV immunization programmes stems from the fact that they present a unique challenge to health systems in sub-Saharan Africa compared with routine childhood immunization programmes. Foremost is the fact that HPV vaccines are not widely accessible through NIPs in most sub-Saharan African countries as compared with other regions of the world (see Figure 1 and Supplementary Table S1).

Three HPV vaccines are currently licenced for use; the four-valent vaccine was licenced in 2006, the bivalent vaccine was licenced in 2007 and the nine-valent vaccine was licenced in 2014 (Herrero *et al.*, 2015; WHO, 2017a). All three prophylactic HPV vaccines have been proven to be safe and effective against persistent HPV infection, which is implicated in a broad spectrum of cancers in both men and women (Lu *et al.*, 2011; Herrero *et al.*, 2015; Garland *et al.*, 2016). The primary focus of this systematic review is HPV vaccination for the prevention of cervical cancer in women. All three HPV vaccines confer immune protection against the HPV oncogenic Types 16 and 18, which are implicated in 70% of all cervical cancers. Cervical cancer, in turn, accounts for 84% of all HPV-associated cancers (WHO, 2017a,b). Given the burden of the disease, the World Health Organization (WHO) recognizes the prevention of cervical cancer as priority and recommends that member states introduce the HPV vaccine into their NIPs (WHO, 2017b). The schedule recommended by WHO is two doses of the HPV vaccine, administered 6 months apart and prioritizing adolescent girls between the ages of 9 and 14 years, prior to sexual debut (WHO, 2017b).

As at December 2019, ∼112 countries and overseas territories had established nationwide HPV immunization programmes (Herrero *et al.*, 2015; LaMontagne *et al.*, 2017; Gallagher *et al.*, 2018; WHO, 2018; 2019). Of this total, only 17 countries are in sub-Saharan Africa (LaMontagne *et al.*, 2017; Gallagher *et al.*, 2018). Another major challenge is the fact that HPV immunization coverage rates in sub-Saharan Africa are reported to be among the lowest (1.2% among 10–20-year-old females) in the world (Bruni *et al.*, 2016). This implies that a substantial proportion of adolescent girls is excluded from the full benefits of the HPV vaccine. This is most concerning, considering that cervical cancer incidence rates in the region are among the highest (31.0 to >40 per 100 000 women) in the world (Louie *et al.*, 2009; de Martel *et al.*, 2017; Arbyn *et al.*, 2020). In addition, cervical cancer is one of the most common causes of cancer-related deaths among women in sub-Saharan Africa (De Vuyst *et al.*, 2013; Arbyn *et al.*, 2020). Evidently, the status of HPV immunization programmes in sub-Saharan Africa is a matter of major public health concern. Expanding access to lifesaving HPV vaccines is a significant challenge for NIPs and health systems in the region (Denny *et al.*, 2012; Sankaranarayanan *et al.*, 2012; Wigle *et al.*, 2013). There is an obvious need for intervention, informed by synthesized evidence on the health systems constraints and facilitators of HPV immunization programmes in sub-Saharan Africa.

## Methods

A qualitative systematic review study was conducted to address the following research question: ‘How do health systems constraints and facilitators impact on the performance of HPV immunization programmes in sub-Saharan Africa?’

A search strategy was developed using key search terms and search term synonyms, which focused on three thematic areas: NIPs, health systems and HPV immunization programmes (full search strategy in Supplementary Table S2). The literature search was limited to studies conducted in sub-Saharan African countries. Using this search strategy, peer-reviewed and grey literature sources were sought through electronic databases such as PubMed, Web of Science, Scopus and EBSCOhost. In addition, supplementary searches for literature sources that may have been missed during the initial electronic search were conducted through Google Scholar as well as organizational websites like WHO (http://www.who.int/en/), GAVI, the Vaccine Alliance (https://www.gavi.org/) and the HPV Information Centre (http://www.hpvcentre.net/). The last date of the primary literature search was August 2018. Updated searches were performed until December 2018. However, this did not yield any additional literature sources relevant to this systematic review.

Only full texts of empirical studies conducted and published between 2008 and 2018 were considered eligible for this systematic review. This is because the past 10 years has been characterized by intensified global efforts to implement HPV immunization programmes, especially in sub-Saharan Africa (Sankaranarayanan *et al.*, 2012; LaMontagne *et al.*, 2017). Reviewing studies conducted within this period therefore enhanced the relevance of the review findings. Eligible studies also included those that used qualitative, quantitative and mixed method study designs. However, modelling studies, reviews, descriptive reports and commentaries reporting on secondary research findings were excluded from this review. In addition, studies published in languages other than English were excluded because of resource constraints. Finally, studies reporting on interventions other than HPV immunization programmes were excluded from the review.

After selection, full texts of eligible studies were critically appraised for the appropriateness of the methods used and the findings reported. Ethical considerations and rigour were also assessed. Evidence of reflexivity, where appropriate, was assessed throughout the texts including authors’ affiliations, research funding and declaration of potential conflict of interest. The quality appraisal was conducted with the assistance of published appraisal tools developed for multiple study designs by the Critical Appraisal Skills Programme (http://www.casp-uk.net/). These appraisal tools have been used previously in other qualitative systematic reviews (Walter *et al.*, 2004; Kane *et al.*, 2007; Chan, 2013).

**Figure 2:**
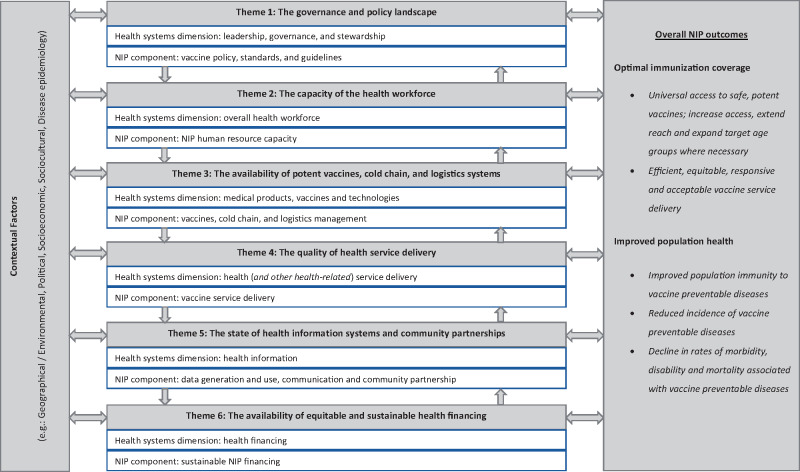
An analytical model for in-depth assessment of the interface between NIPs and health systems.

The analytical approach for this systematic review was guided by an analytical model developed as part of a preliminary scoping review (see Figure 2). While there are several analytical and strategic frameworks proposed for assessing the performance of health systems, these frameworks differ in their starting points, operationalization, as well as process and outcome measures (Arah *et al.*, 2003; Murray and Evans, 2003; Murray and Frenk, 2000; Kruk and Freedman, 2008; WHO, 2011; Mounier-Jack *et al.*, 2014). It is also established that no single framework can holistically evaluate the interaction between disease control interventions and the health system (Mounier-Jack *et al.*, 2014). It is for this reason that an initial scoping review was performed to gain an in-depth understanding of the interaction between NIPs and health systems. This scoping review resulted in an analytical model that integrated the eight components of NIPs as described by Shen *et al.* (2014) and the dimensions of the health system (WHO, 2007). Six cross-cutting themes are addressed in this analytical model, namely, (1) the governance and policy landscape, (2) the capacity of the health workforce, (3) the availability of potent vaccines, cold chain and logistics systems, (4) the quality of health service delivery, (5) the state of health information systems and community partnerships and (6) the availability of equitable and sustainable health financing. The reasoning behind this analytical model is that the interaction among the six cross-cutting themes—which are influenced by underlying contextual factors—will determine whether key NIP targets (like optimal immunization coverage and improved population health) are attained.

Study findings related to health systems constraints and facilitators of HPV immunization programmes were extracted and organized according to the six themes of the model. During the first stage of the analysis process, full texts were imported into Rayyan, a web-based application for systematic reviews, where they were screened for eligibility (Ouzzani *et al.*, 2016). Eligible studies were then coded using inductive and deductive approaches. The codes used were related to the types of health systems constraints and facilitators reported in the study findings. Relevant findings from each study were then extracted and recorded in a database. The extracted data set was then subjected to rigorous thematic analysis with careful consideration for prevailing contextual factors reported for the relevant countries.

Given that majority of sub-Saharan African countries is yet to implement nationwide HPV immunization programmes through their NIPs, we explored outcomes on health systems constraints to implementation. Where studies investigated potential or anticipated health systems constraints and facilitators to the performance of nationwide HPV immunization programmes should they be implemented, these outcomes were also considered. In countries with existing HPV immunization programmes (at the time the studies were conducted), either as part of NIPs or demonstration projects, we also explored findings related to health systems constraints and facilitators to the acceptance and uptake of the HPV vaccine.

## Results

### Characteristics of studies included in this review

**Figure 3:**
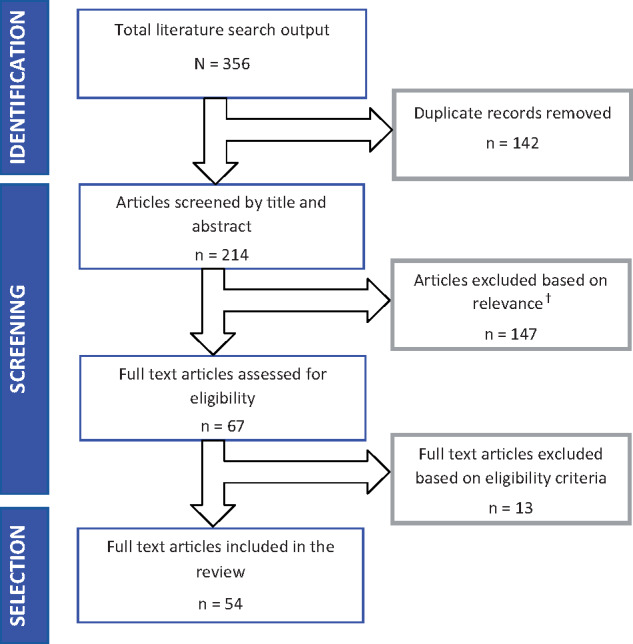
Flowchart of the literature search and selection process. ^†^Reasons for exclusion: study outcomes not relevant for this systematic review (*n* = 68); study design not relevant for this systematic review (*n* = 55); wrong study population (*n* = 13); duplicate records (*n* = 6); full-text unavailable (*n* = 4); and wrong study period (pre-2008) (*n* = 1).

The literature search yielded a total of 356 published records. Overall, 355 of these records were retrieved from electronic databases, while a search in Google Scholar yielded an additional record. No additional unique records were found through organizational websites. Of the total output, 54 full-text articles were found to be eligible for inclusion in the systematic review. Figure 3 shows the screening and selection process for this systematic review. A summary of the quality appraisal of the 54 studies selected for inclusion in this systematic review is provided in Supplementary Table S3. Overall, 52 of the 54 studies met all seven criteria assessed in the appraisal and so were classified as ‘high quality’. The two other studies were classified as low quality and moderate quality (the former not meeting rigour requirements, and the second judged to have inappropriate design, analysis and rigour aspects).

The 54 full-text articles included in this review reported on studies conducted in 20 countries in sub-Saharan Africa, namely, Botswana, Burundi, Cameroon, Côte d’Ivoire, Gambia, Ghana, Kenya, Lesotho, Madagascar, Malawi, Mali, Mozambique, Niger, Nigeria, Rwanda, Senegal, South Africa, Tanzania, Uganda and Zambia (a summary of the data extraction table is provided in Supplementary Table S4). Most articles focused on South Africa (10), Nigeria (8), Kenya (6) and Uganda (6). Where articles reported on findings from multiple countries, only those findings relating to sub-Saharan African countries were extracted. The majority (31) of studies made use of qualitative study designs, while others adopted mixed method (10) or quantitative (10) study designs. A population-based intervention study and a project evaluation were also included in this review (see Table 1).

**Table 1:** Characteristics of studies included in the systematic review

No.	Author (year)	Title	Country	Study design	Availability of HPV vaccine^a^
1	Audu *et al.* (2014)	Awareness and perception of human papilloma virus vaccine among healthcare professionals in Nigeria	Nigeria	Cross-sectional, questionnaire based	Not available
2	Ayissi *et al.* (2012)	Awareness, acceptability and uptake of human papilloma virus vaccine among Cameroonian school-attending female adolescents	Cameroon	Cross-sectional, questionnaire based	Demonstration project
3	Bardají *et al.* (2018)	Awareness of cervical cancer and willingness to be vaccinated against human papillomavirus in Mozambican adolescent girls	Mozambique	Quantitative, cross-sectional	Demonstration project
4	Botha *et al.* (2015)	The Vaccine and Cervical Cancer Screen (VACCS) project: acceptance of human papillomavirus vaccination in a school-based programme in two provinces of South Africa	South Africa	Quantitative	Demonstration project
5	Botwright *et al.* (2017)	Experiences of operational costs of HPV vaccine delivery strategies in Gavi-supported demonstration projects	African countries	Quantitative	Demonstration project
6	Chigbu *et al.* (2017)	The impact of community health educators on uptake of cervical and breast cancer prevention services in Nigeria	Nigeria	Prospective population-based intervention	Demonstration project
7	Coleman *et al.* (2011)	HPV vaccine acceptability in Ghana, West Africa	Ghana	Qualitative, questionnaire based	Available in the private sector only
8	De Groot *et al.* (2017)	Knowledge, attitudes, practices and willingness to vaccinate in preparation for the introduction of HPV vaccines in Bamako, Mali	Mali	Qualitative, household survey	Demonstration project
9	DiAngi *et al.* (2011)	A cross-sectional study of HPV vaccine acceptability in Gaborone, Botswana	Botswana	Cross-sectional, survey	Available in the private sector only
10	Francis *et al.* (2011)	A qualitative analysis of South African women's knowledge, attitudes, and beliefs about HPV and cervical cancer prevention, vaccine awareness and acceptance, and maternal-child communication about sexual health	South Africa	Qualitative	Available in the private sector only
11	Francis *et al.* (2010)	Examining attitudes and knowledge about HPV and cervical cancer risk among female clinic attendees in Johannesburg, South Africa	South Africa	Quantitative	Not available
12	Friedman *et al.* (2014)	Preparing for human papillomavirus vaccine introduction in Kenya: implications from focus-group and interview discussions with caregivers and opinion leaders in Western Kenya	Kenya	Qualitative	Not available
13	Harries *et al.* (2009)	Preparing for HPV vaccination in South Africa: key challenges and opinions	South Africa	Qualitative	Available in the private sector only
14	Hoque (2015)	Acceptability of human papillomavirus vaccination among academics at the University of KwaZulu-Natal, South Africa	South Africa	Qualitative, cross-sectional	Available through NIP
15	Hoque (2016)	Factors influencing the recommendation of the human papillomavirus vaccine by South African doctors working in a tertiary hospital	South Africa	Quantitative, cross-sectional	Available through NIP
16	Hoque *et al.* (2013)	Human papillomavirus vaccination acceptability among female university students in South Africa	South Africa	Qualitative, cross-sectional	Available in the private sector only
17	Hutubessy *et al.* (2012)	A case study using the United Republic of Tanzania: costing nationwide HPV vaccine delivery using the WHO Cervical Cancer Prevention and Control Costing Tool	Tanzania	Quantitative	Not available
18	Kamya *et al.* (2017)	Evaluating global health partnerships: a case study of a Gavi HPV vaccine application process in Uganda	Uganda	Mixed-methods case study	Available through NIP
19	Katz *et al.* (2013)	A qualitative analysis of factors influencing HPV vaccine uptake in Soweto, South Africa among adolescents and their caregivers	South Africa	Qualitative	Available in the private sector only
20	Ladner *et al.* (2012)	Assessment of eight HPV vaccination programs implemented in lowest income countries	Lesotho and Cameroon (non-African countries included)	Mixed methods	Demonstration project
21	Ladner *et al.* (2014)	Performance of 21 HPV vaccination programs implemented in low and middle-income countries, 2009-2013	African countries (non-African LMICs included)	Quantitative	Demonstration project
22	LaMontagne *et al.* (2011)	Human papillomavirus vaccine delivery strategies that achieved high coverage in low- and middle-income countries	Uganda (non-African LMICs included)	Mixed methods, cross-sectional	Demonstration project
23	Levin *et al.* (2013)	Delivery cost of human papillomavirus vaccination of young adolescent girls in Peru, Uganda and Vietnam	Uganda (non-African LMICs included)	Mixed methods	Demonstration project
24	Mabeya *et al*. (2018)	Uptake of three doses of HPV vaccine by primary school girls in Eldoret, Kenya; a prospective cohort study in a malaria endemic setting	Kenya	Cross-sectional, questionnaire based	Demonstration project
25	MacPhail *et al.* (2013)	Using HPV vaccination for promotion of an adolescent package of care: opportunity and perspectives	South Africa	Qualitative, cross-sectional	Available in the private sector only
26	Makwe and Anorlu (2011)	Knowledge of and attitude toward human papillomavirus infection and vaccines among female nurses at a tertiary hospital in Nigeria	Nigeria	Qualitative, cross-sectional	Not available
27	Masika *et al.* (2015)	Knowledge on HPV vaccine and cervical cancer facilitates vaccine acceptability among school teachers in Kitui County, Kenya	Kenya	Mixed methods, cross-sectional	Demonstration project
28	Massey *et al.* (2017)	Human papillomavirus (HPV) awareness and vaccine receptivity among Senegalese adolescents	Senegal	Quantitative, questionnaire based	Demonstration project
29	Moodley *et al.* (2013)	High uptake of Gardasil vaccine among 9–12-year-old schoolgirls participating in an HPV vaccination demonstration project in KwaZulu-Natal province, South Africa	South Africa	Mixed methods	Available in the private sector only
30	Morhason-Bello *et al.* (2015)	Willingness of reproductive-aged women in a Nigerian community to accept human papillomavirus vaccination for their children	Nigeria	Quantitative, multistage household survey	Available in the private sector only
31	Msyamboza *et al.* (2017)	Implementation of a human papillomavirus vaccination demonstration project in Malawi: successes and challenges	Malawi	Mixed methods, cross-sectional	Demonstration project
32	Mugisha *et al.* (2015)	Feasibility of delivering HPV vaccine to girls aged 10 to 15 years in Uganda	Uganda	Qualitative	Demonstration project
33	Ndizeye *et al.* (2018)	Knowledge and practices of general practitioners at district hospitals towards cervical cancer prevention in Burundi, 2015: a cross-sectional study	Burundi	Descriptive, cross-sectional	Not available
34	Ngabo *et al.* (2015)	A cost comparison of introducing and delivering pneumococcal, rotavirus and human papillomavirus vaccines in Rwanda	Rwanda	Quantitative	Available through NIP
35	Odunyemi *et al.* (2018)	Effect of nursing intervention on mothers’ knowledge of cervical cancer and acceptance of human papillomavirus vaccination for their adolescent daughters in Abuja—Nigeria	Nigeria	Quasi-experimental study	Available in the private sector only
36	Ogembo *et al.* (2014)	Achieving high uptake of human papillomavirus vaccine in Cameroon: lessons learned in overcoming challenges	Cameroon	Project evaluation	Demonstration project
37	Okunade *et al.* (2017)	Knowledge and acceptability of human papillomavirus vaccination among women attending the gynaecological outpatient clinics of a university teaching hospital in Lagos, Nigeria	Nigeria	Descriptive, cross-sectional	Available in the private sector only
38	Poole *et al.* (2013)	A cross-sectional study to assess HPV knowledge and HPV vaccine acceptability in Mali	Mali	Qualitative, cross-sectional	Not available
39	Ports *et al.* (2013)	Barriers and facilitators to HPV vaccination: perspectives from Malawian women	Malawi	Qualitative	Not available
40	Quentin *et al.* (2012)	Costs of delivering human papillomavirus vaccination to schoolgirls in Mwanza Region, Tanzania	Tanzania	Mixed methods	Demonstration project
41	Remes *et al.* (2012)	A qualitative study of HPV vaccine acceptability among health workers, teachers, parents, female pupils and religious leaders in northwest Tanzania	Tanzania	Qualitative	Not available
42	Tchounga *et al.* (2014)	Cervical cancer prevention in reproductive health services: knowledge, attitudes and practices of midwives in Côte d’Ivoire, West Africa	Cote d’Ivoire	Qualitative, cross-sectional	Available in the private sector only
43	Torres-Rueda *et al.* (2016)	HPV vaccine introduction in Rwanda: impacts on the broader health system	Rwanda	Mixed methods	Available through NIP
44	Turiho *et al*. (2014)	Effect of school-based human papillomavirus (HPV) vaccination on adolescent girls’ knowledge and acceptability of the HPV vaccine in Ibanda district in Uganda	Uganda	Cross-sectional, mixed methods	Demonstration project
45	Turiho *et al*. (2017)	Perceptions of human papillomavirus vaccination of adolescent schoolgirls in western Uganda and their implications for acceptability of HPV vaccination: a qualitative study	Uganda	Qualitative	Demonstration project
46	Ugwu *et al.* (2013)	Acceptability of human papilloma virus vaccine and cervical cancer screening among female health-care workers in Enugu, Southeast Nigeria	Nigeria	Cross-sectional, questionnaire based	Available in the private sector only
47	Umeh *et al.* (2016)	Mothers’ willingness to pay for HPV vaccines in Anambra state, Nigeria: a cross sectional contingent valuation study	Nigeria	Cross-sectional, survey	Available in the private sector only
48	Urasa and Darj (2011)	Knowledge of cervical cancer and screening practices of nurses at a regional hospital in Tanzania	Tanzania	Descriptive, cross-sectional	Available in the private sector only
49	Venturas and Umeh (2017)	Health professional feedback on HPV vaccination roll-out in a developing country	Zambia	Qualitative	Demonstration project
50	Vermandere *et al.* (2015)	Implementation of an HPV vaccination program in Eldoret, Kenya: results from a qualitative assessment by key stakeholders	Kenya	Qualitative	Demonstration project
51	Vermandere *et al.* (2014)	Determinants of acceptance and subsequent uptake of the HPV vaccine in a cohort in Eldoret, Kenya	Kenya	Qualitative, longitudinal study	Demonstration project
52	Wamai *et al.* (2013)	Awareness, knowledge and beliefs about HPV, cervical cancer and HPV vaccines among nurses in Cameroon: an exploratory study	Cameroon	Qualitative, questionnaire based	Demonstration project
53	Watson-Jones *et al.* (2015)	Access and attitudes to HPV vaccination amongst hard-to-reach populations in Kenya	Kenya	Qualitative	Demonstration project
54	Watson-Jones *et al.* (2012)	Reasons for receiving or not receiving HPV vaccination in primary schoolgirls in Tanzania: a case control study	Tanzania	Qualitative	Demonstration project

a Availability of the HPV vaccine at the time the studies was conducted.

Most studies (33/54) were primarily concerned with assessing knowledge, awareness and acceptability of the HPV vaccine among key populations such as adolescents, parents and caregivers, health workers, teachers and religious leaders (see Table 1). The articles also reported on the availability of the HPV vaccine in the relevant countries at the time each study was conducted. This information tended to change in some countries overtime and presented a unique opportunity to assess the evolution of health systems constraints and facilitators to HPV immunization programmes in these countries. From the overall studies selected for inclusion, the HPV vaccine was reported to be available through the NIPs of South Africa, Rwanda and Uganda. In most countries where the studies were conducted, the HPV vaccine was only available through demonstration projects (see Table 1). Nine articles reported on studies conducted at a time when the HPV vaccine was not available, either through NIPs, demonstration projects or in private health facilities. These studies were mainly focused on determining the feasibility of implementing HPV immunization programmes in the relevant countries by assessing potential health systems constraints and facilitators.

### Health systems constraints and facilitators of HPV immunization programmes in sub-Saharan Africa

We synthesized the available evidence on health systems constraints and facilitators of HPV immunization programmes in sub-Saharan Africa. We considered findings pertaining to health systems constraints and facilitators to (1) HPV vaccine introduction into NIPs, (2) HPV vaccine acceptance and uptake and (3) the overall performance of HPV immunization programmes (include existing nationwide programmes or demonstration projects and future HPV immunization programmes). The findings of this review have been organized and reported under six cross-cutting themes based on the interface between NIPs and health systems.

#### The governance and policy landscape

Where NIPs are concerned, governance functions include the decision- and policy-making processes that go into developing immunization programme-related standards and guidelines, overall programme organization, as well as training and supervision. These functions cut across global, national, sub-national and local or facility levels. Accordingly, this theme explored findings related to leadership, management and stewardship throughout the HPV vaccine policy- and decision-making cascade. The available evidence highlights the importance of clear governance and management structures, the involvement of political champions, the support of policy influencers (including governmental and non-governmental organizations) and the role of strong and inclusive partnerships to the optimal performance of HPV immunization programmes in sub-Saharan Africa (Harries *et al.*, 2009; Ladner *et al.*, 2014; Mugisha *et al.*, 2015; Torres-Rueda *et al.*, 2016; Kamya *et al.*, 2017). For example, prior to the implementation of an HPV immunization programme in South Africa, Harries *et al.* (2009) sought to identify potential barriers and facilitators of such a programme. It was reported that strong collaboration between the national departments of health and education, as well as between the private and public health sectors, was necessary to support and sustain an HPV immunization programme (Harries *et al.*, 2009). These findings were echoed in studies conducted in Rwanda and Uganda where the HPV vaccine had been introduced as part of NIPs (Torres-Rueda *et al.*, 2016; Kamya *et al.*, 2017). In addition, Kamya *et al.* (2017) recognized the role of the First Lady of Uganda as a champion for HPV vaccine introduction in the country. This level of endorsement ensured that the HPV immunization programme remained a priority in the national policy agenda. It was also reported that a diverse and inclusive network of stakeholders from governmental and non-governmental agencies, having past immunization partnership experience and assigned clear roles and responsibilities, was invaluable to a GAVI HPV vaccine application process in Uganda (Kamya *et al.*, 2017). Apart from the national level, the role of governance structures and processes at the health facility level was also identified as critical to HPV immunization programmes. In a study that assessed how HPV vaccine introduction impacted on the Rwandan health system, it was reported that extensive planning and supervision at the health facility level contributed to the successful integration of the HPV vaccine into the NIP (Torres-Rueda *et al.*, 2016).

#### The capacity of the health workforce

The size and competency of the health workforce emerged from the evidence base as priority issues where HPV immunization programmes were concerned. Most studies relating to health workers were conducted at a time when the HPV vaccine was not widely available to the general population, either because it was yet to be introduced in the country or was only available through the private health sector. In a study conducted by Remes *et al.* (2012), health workers identified staff shortages as a potential constraint to implementation of nationwide HPV immunization programme in Tanzania. The major health systems constraint identified in other studies, however, was the inadequate training of the existing workforce in sub-Saharan Africa. Several studies reported that health workers (including doctors, nurses and midwives) exhibited suboptimal knowledge on HPV infection, the pathogenesis and prevention of cervical cancer, the availability, safety and effectiveness of the HPV vaccine and the recommended HPV immunization schedule (Makwe and Anorlu, 2011; Urasa and Darj, 2011; Remes *et al.*, 2012; Wamai *et al.*, 2013; Audu *et al.*, 2014; Tchounga *et al.*, 2014; Hoque, 2016; Venturas and Umeh, 2017; Ndizeye *et al.*, 2018). When the HPV vaccine was not widely available, the proportion of health workers with optimal knowledge about the safety and effectiveness of the HPV vaccine ranged from 13% among nurses in Nigeria (Makwe and Anorlu, 2011) to 55% among general practitioners in Burundi (Ndizeye *et al.*, 2018). However, this increased to 78.9% when the HPV vaccine was available through demonstration projects (Wamai *et al.*, 2013). Health workers’ limited knowledge about the safety of the HPV vaccine was reported to negatively impact on their acceptance of the vaccine for their adolescent daughters as well as their ability to recommend the vaccine to their clients (Makwe and Anorlu, 2011; Wamai *et al.*, 2013; Audu *et al.*, 2014; Hoque, 2016; Venturas and Umeh, 2017). In a study conducted in Cameroon where an HPV vaccine demonstration project was ongoing, 69.7% of nurses surveyed indicated that they often recommended the vaccine to their clients. However, 63.9% of these nurses remained concerned about the potential side effects of the vaccine (Wamai *et al.*, 2013). Poor access to appropriate training was cited as the primary reason for the suboptimal level of knowledge about HPV infection, cervical cancer and HPV immunization, among health workers in sub-Saharan African countries (Urasa and Darj, 2011; Tchounga *et al.*, 2014; Venturas and Umeh, 2017).

Where health workers received adequate training, it was reported that they were capable of providing sound recommendations about the HPV vaccine to clients, thereby positively influencing the acceptance and uptake of the vaccine (Katz *et al.*, 2013; Chigbu *et al.*, 2017; Odunyemi *et al.*, 2018). Additional facilitators of HPV immunization programmes included the involvement of well-trained school health teams as well as community health workers who were reported to play a key role in health promotion and social mobilization for HPV immunization programmes (Moodley *et al.*, 2013; Watson-Jones *et al.*, 2015).

#### The availability of potent vaccines, cold chain and logistics systems

Research evidence on how the availability of the HPV vaccine and the capacity of cold chain and logistics systems (for vaccine delivery, transport and storage) impact on the performance of HPV immunization programmes in sub-Saharan Africa was rather limited. Studies conducted by Okunade *et al.* (2017) and Ugwu *et al.* (2013) addressed the limited availability of the HPV vaccine and how this served as a barrier to the acceptance and uptake of the vaccine in Nigeria. At the time both studies were conducted, the HPV vaccine was not widely available to the general population but was provided through the private health sector at a cost (Ugwu *et al.*, 2013; Okunade *et al.*, 2017). These findings are not surprising as it is well established that lack of access remains one of the key barriers to improved vaccination coverage within sub-Saharan Africa. With regard to cold chain and logistics systems, Torres-Rueda *et al.* (2016) reported that the Rwandan NIP conducted a cold chain inventory to access the capacity of the system prior to the introduction of the HPV vaccine. This formed part of the planning and training implemented in advance of introducing the HPV vaccine into the NIP.

#### The quality of health service delivery

There was much in the literature about the health systems constraints and facilitators to accessing health services that are safe, people centred, integrated and efficient and how this impacts on the performance of HPV immunization programmes in sub-Saharan Africa (see Table 2). Studies exploring the most efficient and effective HPV vaccine delivery models were well represented in the evidence base (LaMontagne *et al.*, 2011; Hutubessy *et al.*, 2012; Ladner *et al.*, 2012; Quentin *et al.*, 2012; Levin *et al.*, 2013; Moodley *et al.*, 2013; Ladner *et al.*, 2014; Ogembo *et al.*, 2014; Watson-Jones *et al.*, 2015; Botwright *et al.*, 2017; Msyamboza *et al.*, 2017). Two main HPV vaccine delivery models, namely, the school-based and health facility-based vaccine delivery strategies were explored. Overall, the findings were consistent, suggesting that adopting a school-based vaccine delivery strategy where eligibility to receive the vaccine was based on school grade or class and not on the age of the recipient was the most effective model (LaMontagne *et al.*, 2011; Ladner *et al.*, 2012; Moodley *et al.*, 2013; Ladner *et al.*, 2014; Mugisha *et al.*, 2015; Msyamboza *et al.*, 2017). This is because the school-based strategy was reported to achieve higher HPV immunization coverage rates compared with the health facility-based strategy (LaMontagne *et al.*, 2011; Ladner *et al.*, 2012, 2014; Mugisha *et al.*, 2015).

**Table 2:** Summary of health systems constraints and facilitators of HPV immunization programmes in sub-Saharan Africa

Theme (*n*^a^)	Health systems constraints	Health systems facilitators
The governance and policy landscape (5)	Weak involvement of Ministries of Education and Finance	Clear governance and management structures Political champions steering and endorsing the agenda for introduction of HPV immunization nationwide Partnerships between all stakeholders—departments of health, finance and education, private and public health sectors Participation of actors with past immunization partnership experience Appropriate supervision, training and planning at the facility level Non-governmental partners playing advocacy and management roles Support of policy influencers
The capacity of the health workforce (17)	Inadequate training of health workers leading to low level of knowledge about HPV infection, cervical cancer and the HPV vaccine Fragile health worker capacity	Adequately trained health workers who can provide sound recommendations to clients about the HPV vaccine Availability of a well-trained school health team Community health workers as source of information and serving as community mobilizers
The availability of potent vaccines, cold chain and logistics systems (3)	Limited availability and accessibility of the HPV vaccine	Conducting national cold chain inventory and ensuring adequate capacity of the cold chain prior to introduction of the HPV vaccine in NIPs
The quality of health service delivery (16)	Adopting age-based or health facility-based community outreach vaccine delivery strategies Health resource constraints Greater resource requirements associated with creating new vaccine delivery infrastructure Logistical challenges of HPV vaccine delivery given the underdevelopment of adolescent health services Long distance to health facilities delivering HPV vaccine Physical barriers to accessing schools, e.g. long distance, unsafe and poor terrain during rainy seasons Poor accessibility of schools and communities for vaccinators due to poor road networks	Implementing mixed vaccine delivery models comprising both grade-based and health facility-based outreach strategies Availability of well-functioning NIPs instilling trust in immunization and improving acceptance and uptake
The state of health information systems and community partnerships (33)	Inadequate sensitization campaigns leading to low level of awareness about the HPV vaccine and the vaccination programme Inadequate engagement with fathers and male teachers Poor access to information about HPV, cervical cancer and HPV vaccine, for individuals living in hard-to-reach communities and among populations with low literacy levels Low level of knowledge about safety and effectiveness of the HPV vaccine Misinformation about the side effects, safety and benefits of the HPV vaccine Misconceptions about other vaccines Negative media reports and interference Exclusive reliance on paper-based vaccine records	Evidence-based health promotion strategies involving intensive community mobilization and sensitization Effective community engagement involving key stakeholders; adolescents, parents (including fathers), teachers, municipal and religious leaders Adequate information, education and communication, especially on vaccine safety and efficacy issues. Prioritizing hard-to-reach communities Consideration for culturally appropriate communication about HPV, cervical cancer and HPV vaccination Adequate communication about all vaccines Strengthening surveillance of adverse events following immunization
The availability of equitable and sustainable health financing (8)	Non-integration of HPV vaccination programmes within existing NIP High financial costs of social mobilization and HPV vaccine delivery as adolescents are not ‘typical’ clients of the health system Adopting an age-based vaccine delivery strategy—less cost-effective High cost of the HPV vaccine Cost of delivering vaccines to adolescents is higher than that of routine childhood immunization	Planning appropriate delivery strategies based on local context—country-specific strategies Intensive investments in community mobilization and sensitization increases vaccine acceptance and uptake Implementing a grade-based delivery strategy—more cost-effective GAVI funding and support Adopting free HPV immunization services

a Numbers in parenthesis represent the number of articles that reported findings related to each theme. Some articles reported on findings pertaining to more than one theme. A detailed description of each of the studies used and the themes they relate to is presented in Supplementary Table S4.

Several challenges to delivering the HPV vaccine through the school-based strategy in sub-Saharan Africa have been documented, however, and these include absenteeism, high school dropout rates among girls and girls transferring out of the district at the time of the immunization programmes (Msyamboza *et al.*, 2017). Physical barriers to accessing the HPV vaccine through schools have also been reported in some hard-to-reach communities in Kenya. These barriers have been associated with non-attendance or delayed enrolment in schools because of long distances (up to 10 km) and safety concerns about possibly encountering wildlife on the way to school (Watson-Jones *et al.*, 2015). In areas like this, where road networks are poorly developed or unsafe, vaccinators have been reported to experience difficulty in accessing schools to provide HPV immunization (Masika *et al.*, 2015). To mitigate these challenges, a mixed vaccine delivery model is highly recommended in the literature and involves coupling the school-based strategy with community outreach immunization campaigns. This mixed vaccine delivery model has been shown to be feasible in sub-Saharan Africa, expanding the reach of HPV immunization programmes, improving adherence to the immunization schedule and scaling up immunization coverage rates (Ladner *et al.*, 2012; Ogembo *et al.*, 2014; Msyamboza *et al.*, 2017).

It was evident from the studies reviewed that the introduction of the HPV vaccine has exposed gaps in the development of adolescent and school health services in sub-Saharan Africa. This has been considered in the evidence base as a constraint to HPV immunization programmes because of the logistical challenges to providing health services to a previously underserved adolescent population and the greater resource requirements associated with creating new vaccine delivery infrastructure (Harries *et al.*, 2009; Remes *et al.*, 2012; Ngabo *et al.*, 2015). A study conducted in South Africa by MacPhail *et al.* (2013) also identified gaps in the appropriate integration of the HPV immunization programme with other adolescent health services such as sex education, screening and preventive services, assistance with substance abuse and provision of other adolescent vaccines.

#### The state of health information systems and community partnerships

Three main dimensions were explored under this theme; (1) health information, education and communication about HPV infection, cervical cancer and HPV immunization; (2) community partnerships during HPV immunization programmes; and (3) the generation and use of surveillance and immunization data. After reviewing the evidence base, the state of health information systems and community partnerships emerged as the most researched theme where HPV immunization programmes in sub-Saharan Africa are concerned (see Table 2).

Several studies were conducted to assess the level of awareness about HPV infection, cervical cancer and HPV immunization among general populations in sub-Saharan Africa. Study participants included key stakeholders like adolescents, women of reproductive age, parents or caregivers, teachers, opinion leaders, religious leaders and university students and academics (Francis *et al.*, 2010; Coleman *et al.*, 2011; DiAngi *et al.*, 2011; Francis *et al.*, 2011; Ayissi *et al.*, 2012; Remes *et al.*, 2012; Watson-Jones *et al.*, 2012; Hoque *et al.*, 2013; MacPhail *et al.*, 2013; Poole *et al.*, 2013; Ports *et al.*, 2013; Turiho *et al.*, 2014; Vermandere *et al.*, 2014; Botha *et al.*, 2015; Hoque, 2015; Masika *et al.*, 2015; Morhason-Bello *et al.*, 2015; Vermandere *et al.*, 2015; De Groot *et al.*, 2017; Massey *et al.*, 2017; Okunade *et al.*, 2017; Turiho *et al.*, 2017; Bardají *et al.*, 2018). Majority of these studies found that the level of awareness among the general population was rather limited. With the exception of university academics, the proportion of the general population found to be aware of HPV infection, cervical cancer and the HPV vaccine was generally low and ranged from 8.6% to 36.5%, 61% to 87% and 0% to 40%, respectively (Francis *et al.*, 2010; Coleman *et al.*, 2011; DiAngi *et al.*, 2011; Remes *et al.*, 2012; Ports *et al.*, 2013; Friedman *et al.*, 2014; De Groot *et al.*, 2017; Massey *et al.*, 2017; Okunade *et al.*, 2017). In comparison, the highest level of awareness about HPV (100%) and cervical cancer (96%) was recorded among university academics (Hoque, 2015).

**Figure 4:**
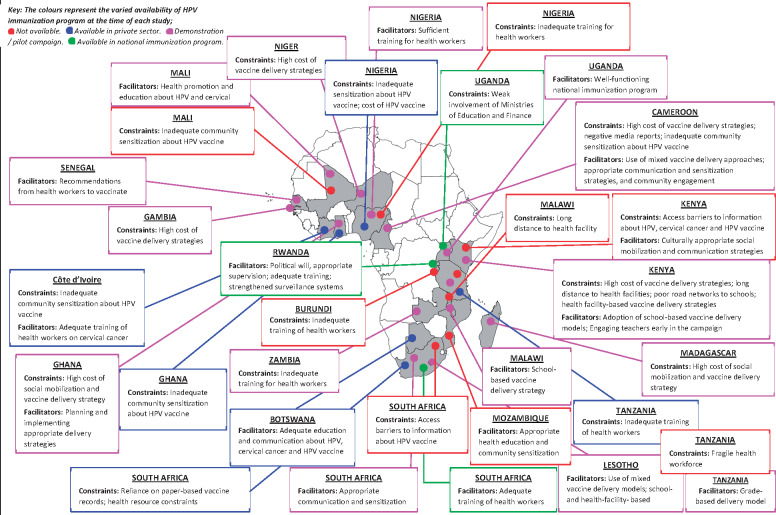
Summary of health systems constraints and facilitators of HPV immunization programmes in sub-Saharan Africa.

The low level of awareness demonstrated among the general population was reported to fuel misconceptions about the safety and benefits of the HPV vaccine. Some misconceptions about the potential side effects of the vaccine included infertility and death, while some feared that introduction of the vaccine would promote promiscuity and early sexual debut among adolescent girls (Watson-Jones *et al.*, 2012; Ogembo *et al.*, 2014; Morhason-Bello *et al.*, 2015). Other studies also reported that some participants believed that the HPV vaccine would prevent HIV and pregnancy, or even safeguard the fertility of adolescent girls (Vermandere *et al.*, 2015; Turiho *et al.*, 2017). Ultimately, low level of awareness was a major constraint to the acceptance and uptake of the HPV vaccine in sub-Saharan Africa (Vermandere *et al.*, 2014). When intensive social mobilization and community sensitization interventions were implemented to provide culturally appropriate information about the risks of cervical cancer and the safety and effectiveness of the HPV vaccine (often as part of vaccine demonstration projects; see Figure 4), an increase in vaccine acceptance and uptake was reported (Coleman *et al.*, 2011; DiAngi *et al.*, 2011; Ayissi *et al.*, 2012; Ports *et al.*, 2013; Turiho *et al.*, 2014; Botha *et al.*, 2015; Hoque, 2015; Masika *et al.*, 2015; Vermandere *et al.*, 2015; De Groot *et al.*, 2017; Bardají *et al.*, 2018). Participants also identified health workers as their most trusted source of information about the HPV vaccine (Ayissi *et al.*, 2012; Ports *et al.*, 2013; Massey *et al.*, 2017). Engaging key stakeholders like adolescents (both males and females), parents (including fathers) or caregivers, municipal and religious leaders and school teachers, at the onset of HPV immunization programmes, was considered as a major facilitator to vaccine acceptance and uptake (Ports *et al.*, 2013; Ogembo *et al.*, 2014; Masika *et al.*, 2015; Vermandere *et al.*, 2015).

Very few studies reported on how the generation and use of surveillance and immunization data (including immunization coverage data) impacted on the performance of HPV immunization programmes in sub-Saharan Africa. Where studies explored this dimension, the evidence found was rather limited. For example, a study conducted to assess the capacity of school health teams to carry out future HPV immunization in schools within the KwaZulu-Natal province of South Africa found that the reliance on paper-based vaccine records would be a major constraint to real-time monitoring and evaluation of the immunization programme. To mitigate this challenge, the use of electronic data capturing methods was recommended (Moodley *et al.*, 2013). In addition to this, Torres-Rueda *et al.* (2016) highlighted the importance of strengthening the surveillance of adverse events following immunization in Rwanda where a nationwide HPV immunization programme was already in existence (see Figure 4).

#### The availability of equitable and sustainable health financing

The final theme examined national health financing mechanisms in sub-Saharan Africa, including the availability of equitable and sustainable financing mechanisms for HPV immunization programmes. Research evidence on this theme was found to be limited. None of the studies included in the review addressed any dedicated financing mechanisms earmarked for the introduction and sustainable delivery of the HPV vaccine. Instead, the available evidence on financing was mainly focused on determining the economic and financial costs of social mobilization campaigns and HPV vaccine delivery models (Hutubessy *et al.*, 2012; Quentin *et al.*, 2012; Levin *et al.*, 2013; Ngabo *et al.*, 2015; Botwright *et al.*, 2017).

With the exception of South Africa, HPV vaccine procurement costs were reported to be largely covered by external funding sources (from organizations like GAVI, Program for Appropriate Technology in Health and the Gardasil Access Program), in countries where the HPV immunization programme had been implemented as part of demonstration projects or NIPs (Ladner *et al.*, 2014; Mugisha *et al.*, 2015; Ngabo *et al.*, 2015; Botwright *et al.*, 2017). In most cases, national governments are expected to co-finance the cost of delivering the vaccine. However, this was a major constraint to implementing HPV immunization programmes, given that the financial cost of introducing and delivering the HPV vaccine was significantly higher than that of routine childhood vaccines (Levin *et al.*, 2013; Ngabo *et al.*, 2015). Reasons given for this included the fact that the infrastructure for delivering vaccines to adolescents was largely underdeveloped prior to the introduction of the HPV vaccine. As such financial costs were reported to be higher in countries that could not leverage existing immunization infrastructure and had greater resource requirements (Ngabo *et al.*, 2015; Botwright *et al.*, 2017).

It was reported that a substantial cost component was required to train health workers, cover per diems, organize effective social mobilization and community sensitization campaigns and deliver the vaccine through the school-based strategy (Hutubessy *et al.*, 2012; Levin *et al.*, 2013; Ngabo *et al.*, 2015; Botwright *et al.*, 2017). With regard to the school-based vaccine delivery strategy, adopting an age-based eligibility approach was found to be a constraint to HPV immunization programmes as it was less cost-effective compared with the grade-based approach (Quentin *et al.*, 2012). However, delivering the HPV vaccine through health facilities incurred lower economic costs compared with the school-based strategy but achieved lower immunization coverage rates (Levin *et al.*, 2013). To offset the cost of delivering the HPV vaccine, some countries were reported to charge a fee for immunization, while in other countries, the vaccine was only available through the private health sector at unaffordable costs (Ugwu *et al.*, 2013; Ogembo *et al.*, 2014; Umeh *et al.*, 2016; Okunade *et al.*, 2017; Odunyemi *et al.*, 2018). In these countries, socioeconomic constraints to accessing the HPV vaccine negatively impacted on vaccine acceptance and uptake.

## Discussion

There is growing consensus that the success of key health programmes in achieving improved population health is highly dependent on the performance of their health systems (Travis *et al.*, 2004; Balabanova *et al.*, 2010). In this regard, previous systematic reviews have assessed the health systems constraints and facilitators to the effective performance of several health programmes, such as those targeted at HIV anti-retroviral treatment, prevention of mother-to-child transmission of HIV and the prevention and control of breast cancer, as well as other chronic diseases (Colvin *et al.*, 2014; Bowser *et al.*, 2017; Watt *et al.*, 2017). The primary intent of these reviews is to build up the evidence base required to inform health systems strengthening efforts. This has been spurred by the realization that strong health systems are able to withstand acute shocks or avoid them altogether, to provide health programmes with the support they require to run effectively (Travis *et al.*, 2004; Balabanova *et al.*, 2010; Hanvoravongchai *et al.*, 2011; Wang *et al.*, 2013). To date, no rigorous systematic reviews exist on the health systems constraints and facilitators of NIPs, especially from the sub-Saharan African region.

At the World Health Assembly held in May 2012, 194 member states endorsed the Global Vaccine Action Plan. One of the visions of the Global Vaccine Action Plan is to advance universal access to immunization between 2011 and 2020 (WHO, 2013b; WHO, 2015). To date, regional immunization efforts have been heavily focused on routine childhood immunization programmes, which have achieved remarkable public health success within the subcontinent (Arevshatian *et al.*, 2007; Machingaidze *et al.*, 2013). However, if sub-Saharan Africa is to have a chance of achieving the vision of the Global Vaccine Action Plan beyond 2020, countries will have to scale up the performance of existing immunization programmes, introduce and improve the uptake of underused vaccines and extend access to lifesaving vaccines to underserved and hard-to-reach populations (WHO, 2013b; Nanni *et al.*, 2017; Bonner *et al.*, 2018). Scaling up the HPV immunization programmes, which target the adolescent population, presents a unique opportunity to contribute to attaining the vision of the Global Vaccine Action Plan beyond 2020. In this regard, Nanni *et al.* (2017) advocate for ‘global consensus, political will, policies, global and country infrastructure and financing mechanisms’ to accelerate universal access to the HPV vaccine for adolescent populations in low- and middle-income regions like sub-Saharan Africa. To facilitate this, it is important to provide decision-makers with robust systematic evidence of what works (and what does not work) for HPV immunization programmes in sub-Saharan Africa, especially where health systems are concerned.

The findings of this systematic review are consistent with the recommendations made by Nanni *et al.* (2017). In sub-Saharan African countries like Uganda where national governments have strongly endorsed the HPV vaccine and have made concerted efforts through national policy to ensure that the vaccine is widely available to the population, HPV immunization programmes have been successfully implemented as part of NIPs and vaccine acceptance and uptake has significantly improved (Mugisha *et al.*, 2015; Kamya *et al.*, 2017). Global infrastructure for HPV immunization programmes has typically come in the form of external funding, and expert and technical support from organizations like GAVI, Program for Appropriate Technology in Health and the Gardasil Access Program (Ladner *et al.*, 2014; Mugisha *et al.*, 2015; Ngabo *et al.*, 2015; Botwright *et al.*, 2017). The support of these global partners has been instrumental in facilitating the introduction of HPV immunization programmes in sub-Saharan Africa, either through demonstration projects or NIPs (Oluwole and Kraemer, 2013; Ladner *et al.*, 2014; Gallagher *et al.*, 2017b, 2018). While these health systems facilitators (strong political endorsement, clear governance structures and partnerships with global partners) have achieved some progress for HPV immunization programmes in sub-Saharan Africa, the region continues to fall short in meeting global targets for implementing HPV immunization programmes. In addition to this, HPV immunization coverage rates in sub-Saharan Africa are recorded among the lowest in the world (Bruni *et al.*, 2016; LaMontagne *et al.*, 2017; Gallagher *et al.*, 2017a, 2018). These persistent challenges may be the result of significant health systems constraints in sub-Saharan Africa, as has been suggested previously (Bello *et al.*, 2011; Wigle *et al.*, 2013; SAGE, 2016).

The most recurrent health systems constraint identified by this systematic review was the low level of knowledge and awareness on HPV infection, the risk of cervical cancer and the safety and effectiveness of the HPV vaccine, among general populations in sub-Saharan Africa. This finding is highly relevant given the well-established link between levels of knowledge, awareness and attitudes towards the HPV vaccine, and vaccine acceptance and uptake (Garcini *et al.*, 2012; Kessels *et al.*, 2012; Hopkins and Wood, 2013; Maseko *et al.*, 2015). The gap identified in the level of knowledge and awareness among the general population is not unique to the sub-Saharan African context and has been reported in other parts of the world where HPV immunization programmes have long been in existence (Hendry *et al.*, 2013; Cartmell *et al.*, 2018). Nonetheless, this calls into question the effectiveness of existing HPV vaccine communication strategies in sub-Saharan Africa. Key stakeholders, including adolescents and their caregivers, depend on reliable communication to make informed decisions about lifesaving HPV vaccines and take ownership of their health (Bonner *et al.*, 2018). Evidently, there is a need to strengthen health systems in sub-Saharan Africa by reinforcing the role of health promotion activities, especially where HPV immunization programmes are concerned. Where intensified social mobilization and community sensitization campaigns have been implemented, this was shown to increase the demand for the HPV vaccine. In addition to this, the importance of designing culturally appropriate communication strategies, which are also accessible to populations living in hard-to-reach communities, was emphasized in the literature (Friedman *et al.*, 2014; Watson-Jones *et al.*, 2015).

The limited capacity of health workers was identified as another major health systems constraint to the performance of HPV immunization programmes in sub-Saharan Africa. Overall, health workers demonstrated suboptimal levels of knowledge and awareness about HPV infection, the risk of cervical cancer and the safety and effectiveness of the HPV vaccine. These findings are consistent with those reported in other resource limited settings (Nganwai *et al.*, 2008; Ali *et al.*, 2010; Chawla *et al.*, 2016). In contrast, reports from some high resource settings indicate high levels of knowledge and awareness about HPV infection, cervical cancer and the HPV vaccine, among health workers (Rosen *et al.*, 2018). This is a matter of major public health concern in sub-Saharan Africa, given that cadres like nurses and community health workers are at the frontline of HPV immunization programmes and have been identified as the most trusted source of information on HPV immunization (Ayissi *et al.*, 2012; Ports *et al.*, 2013; Watson-Jones *et al.*, 2015; Massey *et al.*, 2017). There is a need, therefore, to reinforce the capacity of health workers in sub-Saharan Africa. This can be achieved through continued training and education as immunization programmes evolve, to ensure that health workers can provide sound and up-to-date vaccine recommendations to their clients.

Historically, adolescents have not been the ‘typical’ client base of most health systems. However, with the introduction of HPV immunization programmes, this notion is beginning to change (Ladner *et al.*, 2012; MacPhail *et al.*, 2013; Nanni *et al.*, 2017). This systematic review has found that poorly developed adolescent and school health services present a significant challenge to effectively delivering HPV vaccines in sub-Saharan Africa. This is further compounded by weak country infrastructure, including underdeveloped and unsafe road networks, as well as geographically inaccessible schools and health facilities, which create physical barriers to reaching target populations with the HPV vaccine (Masika *et al.*, 2015; Watson-Jones *et al.*, 2015; Msyamboza *et al.*, 2017). A review by Sankaranarayanan *et al.* (2012), which sought to address the infrastructure requirements for HPV immunization programmes in sub-Saharan Africa, found that, although leveraging the existing NIP resources (e.g. human resources and cold chain and logistics systems) could improve the performance of HPV immunization programmes, greater investments in infrastructure are still needed. This calls for intersectoral collaboration within national governments, involving relevant Ministries like Health, Education and Finance. Improving adolescent and school health services to support HPV immunization programmes and administration of other adolescent vaccines also deserves particular attention in the health policy agenda in sub-Saharan Africa (Nanni *et al.* 2017).

Finally, this review found that significant financial constraints impact on the introduction of HPV immunization programmes and the delivery of the vaccine to adolescents in sub-Saharan Africa. The HPV vaccine is more costly than other routine childhood vaccines. In addition, the HPV vaccine delivery models incur higher financial and economic costs when compared with routine childhood immunization programmes (Levin *et al.*, 2013; Ngabo *et al.*, 2015). These financial barriers are intensified when considered in light of unique sub-Saharan African contextual issues, such as the limited health budgets of some national governments and the high burden of diseases, which has given rise to multiple health programmes (such as those targeting malaria, HIV, and other vaccine-preventable diseases) competing for the limited resources (Bello *et al.*, 2011). Altogether, these financial constraints significantly obstruct the widespread implementation of HPV immunization programmes in sub-Saharan Africa (Gallagher *et al.*, 2018). While substantial investments have been made by global partners, co-financing commitments make HPV vaccine introduction unaffordable for some national governments in sub-Saharan Africa (Gallagher *et al.*, 2018). Increasing national investments in HPV immunization programmes will require reforms in health policy, although this will have to be informed by country-specific evidence on the resource needs and the most effective and sustainable financing mechanisms to be adopted.

From 2020, other sub-Saharan African countries will be looking to introduce HPV immunization programmes nationwide. While this will be an impressive move towards expanding access to lifesaving HPV vaccines within the region, it is important that key decision-makers take into consideration the health systems constraints and facilitators presented in this review. Countries could draw on the health systems facilitators to well-performing HPV immunization programmes in developing country-specific standards and guidelines for their immunization programmes. The findings on health systems constraints also serve as a set of ‘lessons learned’ for HPV immunization programmes in sub-Saharan Africa so far and could be used to inform interventions to scale up the performance of existing immunization programmes or to guide the implementation of new HPV immunization programmes where necessary.

Although this systematic review assessed health systems constraints and facilitators to HPV immunization programmes, the findings have significant relevance for scaling up NIPs in general. The major health systems constraints and facilitators pertaining to service delivery, the health workforce, vaccine communication and community partnerships, as well as governance and policy, are applicable to almost all services delivered through NIPs. For example, barriers to accessing vaccines due to ineffective service delivery, staff shortages, lack of awareness about vaccines, weak engagement with key stakeholders, as well as poor governance and policy structures have been suggested to negatively impact on the performance of NIPs in most LMICs, and the findings of this review are in support of this (Shen *et al.*, 2014; SAGE, 2016; Mihigo *et al.*, 2017). What has not been sufficiently addressed in the existing literature is how to overcome these constraints and strengthen broader health systems to support optimal NIP performance. In addition to this, detailed considerations of the cost-effectiveness of strengthening the broader health system in the anticipation of scaling up NIP performance are scarce. This systematic review provides evidence on health systems facilitators, which can be leveraged in developing interventions to mitigate some of the system-wide barriers to scaling up NIPs. It is important to caution, however, that HPV immunization programmes have some unique characteristics when compared with other NIP services. First, HPV immunization programmes are targeted at the adolescent population while most NIP services have typically been focused on infants and children. Second, the vaccine delivery models for HPV immunization programmes are also different from those typically used in other NIP services. As such certain variations exist in the health systems functions needed to support HPV immunization programmes compared with routine childhood immunization services, as discussed previously. Despite this, NIPs are beginning to evolve towards a ‘life-course approach’ to immunization by expanding target age groups and extending immunization services to adolescents and adults who have been previously underserved (Mehta *et al.*, 2014; Langley, 2015). In this regard, the findings of this systematic review could be useful for informing health systems strengthening initiatives to support the expanding scope of NIPs, especially in sub-Saharan Africa.

Limitations of this systematic review include the geographical restriction to sub-Saharan Africa. The findings and recommendations of the review may not be generalizable to parts of the world that do not share similar contexts with countries in sub-Saharan Africa. The language restriction applied during the search strategy is another limitation as relevant studies conducted and published from non-Anglophone countries may have been missed. While this could imply that evidence on fragile populations with the highest burden of disease and greatest need for intervention may have been excluded, it is important to caution that this is reflective of the broader field and scope of the literature. It is also worth noting that most studies on knowledge and awareness were based on self-reporting and might be subject to reporting bias. Interpretation of the findings of this systematic review may have been limited to the analytical approach used, which was guided by an analytical model developed as part of a preliminary scoping review. Finally, the use of HPV immunization programmes as a proxy for NIPs may not give a comprehensive assessment given some of the programmatic differences with other routine childhood immunization programmes. The generalizability of the findings of this systematic review for other NIP services may therefore require further investigation, adapting and using the six cross-cutting themes on the interface between NIPs and health systems.

## Conclusion

This systematic review provides evidence of how NIPs and health systems interact by reporting on the health systems constraints and facilitators of NIPs in sub-Saharan Africa. In addition, the findings show how these system-wide constraints and facilitators impact on the performance of NIPs. There is evidence to suggest that NIPs in sub-Saharan Africa have surmounted significant health systems constraints and have achieved notable public health success. This success can be attributed to strong political endorsement for vaccines, clear governance structures and effective collaboration with global partners. Despite this, important health systems constraints persist and could derail further progress if not addressed through health systems strengthening efforts. Gaps in the evidence base pertaining to cold chain and logistics systems, data generation and use, as well as national financing mechanisms needed to sustain NIPs in sub-Saharan Africa were also identified. This calls for an expansion of the research agenda, not only to address these gaps, but also to continuously evaluate health systems constraints and facilitators of NIPs in sub-Saharan Africa. The findings of this review are relevant to ongoing health systems strengthening initiatives in sub-Saharan Africa. By enhancing our understanding of what works—‘and does not work’—for NIPs, health systems strengthening initiatives could be better designed to adequately respond to the burden of vaccine-preventable diseases in sub-Saharan Africa.

## Supplementary data

Supplementary data are available at *Health Policy and Planning* online.

*Conflict of interest statement*. None declared.

*Ethical approval.* No ethical approval was required for this study.

## Supplementary Material

czaa017_Supplementary_Data
