# Supplementary material for: Health systems constraints and facilitators of human papillomavirus immunization programmes in sub-Saharan Africa: a systematic review
Source: Health Policy Plan. 2020 May 3;35(6):701–17. doi: 10.1093/heapol/czaa017 (PMC7294244; doi:10.1093/heapol/czaa017)
Supplement: czaa017_Supplementary_Data [file czaa017_supplementary_data.docx]

Supplementary Table 1: Human Papillomavirus Vaccine Introduction Worldwide

| **Country / Territory** | | **Income** | | | **Introduction of HPV vaccine in NIP** | | **Year of Introduction** | | **Comment** |
| --- | --- | --- | --- | --- | --- | --- | --- | --- | --- |
| **East Asia and Pacific** | | | | | | | | | |
| American Samoa | | UMIC | | No | | |  | | Partial introduction in parts of the country in 2013 |
| Australia | | HIC | | Yes | | | 2007 | |  |
| Brunei Darussalam | | HIC | | Yes | | | 2012 | |  |
| Cambodia | | LMIC | | No | | |  | | HPV vaccine demonstration project launched in January 2017 with intent to scale-up to a national program in 2019 |
| China | | UMIC | | No | | |  | | Approved for use by the Chinese Food and Drug Administration in 2016 |
| Cook Islands | |  | | Yes | | | 2011 | |  |
| Fiji | | UMIC | | Yes | | | 2013 | |  |
| French Polynesia | | HIC | |  | | |  | | No data found on HPV immunization program |
| Guam | | HIC | | Yes | | | 2012 | |  |
| Hong Kong SAR, China | | HIC | | No | | |  | | HPV vaccine demonstration project launched in October 2016 for 3 years |
| Indonesia | | LMIC | | No | | |  | | HPV vaccine demonstration project launched in 2016 |
| Japan | | HIC | | No | | |  | | HPV vaccine introduced nationwide in 2011, but suspended in 2013 |
| Kiribati | | LMIC | | Yes | | | 2011 | |  |
| Korea, Democratic People's Republic | | LIC | | No | | |  | | No nationwide HPV immunization program |
| Korea, Republic | | HIC | | Yes | | | 2016 | |  |
| Lao PDR | | LMIC | | No | | |  | | HPV demonstration project launched in October 2013 |
| Macao SAR, China | | HIC | | Yes | | | 2013 | |  |
| Malaysia | | UMIC | | Yes | | | 2010 | |  |
| Marshall Islands | | UMIC | | Yes | | | 2008 | |  |
| Micronesia, Federated States | | LMIC | | Yes | | | 2009 | |  |
| Mongolia | | LMIC | | No | | |  | | HPV vaccine demonstration project rolled out between 2009 to 2013 |
| Myanmar | | LMIC | | No | | |  | | No nationwide HPV immunization program. Announced for 2020 |
| Nauru | | UMIC | | No | | |  | | No nationwide HPV immunization program |
| New Caledonia | | HIC | | Yes | | | 2011 | |  |
| New Zealand | | HIC | | Yes | | | 2008 | |  |
| Niue | |  | | Yes | | |  | | No data found on year of HPV vaccine introduction |
| Northern Mariana Islands | | HIC | | Yes | | | 2008 | |  |
| Palau | | HIC | | Yes | | | 2009 | |  |
| Papua New Guinea | | LMIC | | No | | |  | | HPV vaccine demonstration project launched in 2017 |
| Philippines | | LMIC | | Yes | | | 2016 | |  |
| Samoa | | UMIC | | No | | |  | | No nationwide HPV immunization program |
| Singapore | | HIC | | Yes | | | 2010 | |  |
| Solomon Islands | | LMIC | | No | | |  | | HPV vaccine demonstration project launched in 2015 |
| Taiwan, China | | HIC | | No | | |  | | HPV vaccine demonstration project launched in 2008. Introduction announced for November 2018 |
| Thailand | | UMIC | | Yes | | | 2017 | | Introduced in August 2017 |
| Timor-Leste | | LMIC | | No | | |  | | No nationwide HPV immunization program |
| Tonga | | UMIC | | No | | |  | | No nationwide HPV immunization program |
| Tuvalu | | UMIC | | No | | |  | | No nationwide HPV immunization program |
| Vanuatu | | LMIC | | Yes | | | 2015 | |  |
| Vietnam | | LMIC | | No | | |  | | HPV vaccine demonstration project rolled-out between in 2008 to 2010 |
| Wallis and Futuna | |  | | No | | |  | | No nationwide HPV immunization program |
| **Europe and Central Asia** | | | | | | | | | |
| Albania | | UMIC | | No | | |  | | No nationwide HPV immunization program |
| Andorra | | HIC | | Yes | | | 2014 | |  |
| Armenia | | UMIC | | No | | |  | | HPV vaccine demonstration project launched in 2017 for 2 years |
| Austria | | HIC | | Yes | | | 2014 | |  |
| Azerbaijan | | UMIC | | No | | |  | | No nationwide HPV immunization program |
| Belarus | | UMIC | | No | | |  | | No nationwide HPV immunization program |
| Belgium | | HIC | | Yes | | | 2007 | |  |
| Bosnia and Herzegovina | | UMIC | | No | | |  | | No nationwide HPV immunization program |
| Bulgaria | | UMIC | | Yes | | | 2012 | |  |
| Croatia | | HIC | | Yes | | | 2016 | |  |
| Cyprus | | HIC | | Yes | | | 2016 | |  |
| Czech Republic | | HIC | | Yes | | | 2012 | |  |
| Denmark | | HIC | | Yes | | | 2009 | |  |
| Estonia | | HIC | | Yes | | | 2018 | |  |
| Faroe Islands | | HIC | |  | | |  | | No data found on HPV immunization program |
| Finland, Republic | | HIC | | Yes | | | 2013 | |  |
| France | | HIC | | Yes | | | 2007 | |  |
| French Guiana | |  | |  | | |  | | Commercialized since 2007 following recommendation in France |
| Georgia | | LMIC | | No | | |  | | HPV vaccine demonstration project was launched |
| Germany | | HIC | | Yes | | | 2007 | |  |
| Gibraltar | | HIC | | Yes | | | 2008 | |  |
| Greece | | HIC | | Yes | | | 2008 | |  |
| Greenland | | HIC | | Yes | | | 2008 | |  |
| Guernsey | |  | | Yes | | | 2015 | | May have been introduced in 2015 |
| Hungary | | HIC | | Yes | | | 2014 | |  |
| Iceland | | HIC | | Yes | | | 2011 | |  |
| Ireland | | HIC | | Yes | | | 2010 | |  |
| Isle of Man | | HIC | | Yes | | | 2009 | |  |
| Italy | | HIC | | Yes | | | 2007 | |  |
| Bailiwick of Jersey | |  | | Yes | | | 2008 | |  |
| Kazakhstan | | UMIC | | Yes | | | 2013 | |  |
| Kosovo | | LMIC | |  | | |  | | No data found on HPV immunization program |
| Kyrgyz Republic | | LMIC | | No | | |  | | No nationwide HPV immunization program |
| Latvia | | HIC | | Yes | | | 2010 | |  |
| Liechtenstein | | HIC | | Yes | | | 2007 | | Approved for use in 2007 |
| Lithuania | | HIC | | Yes | | | 2016 | |  |
| Luxembourg | | HIC | | Yes | | | 2008 | |  |
| Macedonia, FYR | | UMIC | | Yes | | | 2009 | |  |
| Moldova | | LMIC | | Yes | | | 2012 | | Introduced in November 2017 |
| Monaco | | HIC | | Yes | | | 2011 | |  |
| Montenegro | | UMIC | | No | | |  | | No nationwide HPV immunization program. The HPV vaccine not been registered for use as at March 2018 |
| Netherlands | | HIC | | Yes | | | 2010 | |  |
| Norway | | HIC | | Yes | | | 2009 | |  |
| Poland | | HIC | | No | | |  | | No nationwide HPV immunization Program. The vaccine has been introduced in some territorial self-government Units |
| Portugal | | HIC | | Yes | | | 2008 | |  |
| Romania | | UMIC | | No | | |  | | No nationwide HPV immunization program. Voluntary immunization campaigns held in 2008 to 2010, but campaigns were discontinued due to low acceptance and uptake |
| Russian Federation | | UMIC | | Yes | | | 2009 | |  |
| San Marino | | HIC | | Yes | | | 2008 | |  |
| Serbia | | UMIC | | No | | |  | | No nationwide HPV immunization program |
| Slovak Republic | | HIC | | Yes | | | 2014 | |  |
| Slovenia | | HIC | | Yes | | | 2009 | |  |
| Spain | | HIC | | Yes | | | 2007 | |  |
| Sweden | | HIC | | Yes | | | 2010 | |  |
| Switzerland | | HIC | | Yes | | | 2008 | |  |
| Tajikistan | | LIC | | No | | |  | | No nationwide HPV immunization program |
| Turkey | | UMIC | | No | | |  | | No nationwide HPV immunization program |
| Turkmenistan | | UMIC | | Yes | | | 2016 | |  |
| Ukraine | | LMIC | | No | | |  | | No nationwide HPV immunization program |
| United Kingdom | | HIC | | Yes | | | 2008 | |  |
| Uzbekistan | | LMIC | | Yes | | | 2015 | |  |
| **Latin America and the Caribbean** | | | | | | | | | |
| Anguilla | | HIC | | Yes | | 2016 | | Introduced in April 2016 | |
| Antigua and Barbuda | | HIC | | Yes | | 2018 | | Nationwide HPV immunization begun in July 2018 | |
| Argentina | | HIC | | Yes | | 2011 | |  | |
| Aruba | | HIC | | No | |  | | No nationwide HPV immunization program | |
| Bahamas, The | | HIC | | Yes | | 2015 | |  | |
| Barbados | | HIC | | Yes | | 2014 | |  | |
| Belize | | UMIC | | Yes | | 2016 | |  | |
| Bolivia | | LMIC | | No | |  | | HPV vaccine demonstration projects rolled-out between 2009 - 2011 | |
| Brazil | | UMIC | | Yes | | 2014 | |  | |
| British Virgin Islands | | HIC | |  | |  | | No data found on HPV immunization program | |
| Cayman Islands | | HIC | | Yes | | 2012 | |  | |
| Chile | | HIC | | Yes | | 2014 | |  | |
| Colombia | | UMIC | | Yes | | 2012 | |  | |
| Costa Rica | | UMIC | | No | |  | | No nationwide HPV immunization program | |
| Cuba | | UMIC | | No | |  | | No nationwide HPV immunization program | |
| Dominica | | UMIC | | No | |  | | No nationwide HPV immunization program | |
| Dominican Republic | | UMIC | | No | |  | | HPV immunization campaign rolled-out in April 2017 | |
| Ecuador | | UMIC | | Yes | | 2015 | |  | |
| El Salvador | | LMIC | | No | |  | | No nationwide HPV immunization program | |
| Grenada | | UMIC | | No | |  | | No nationwide HPV immunization program | |
| Guatemala | | UMIC | | No | |  | | No nationwide HPV immunization program | |
| Guyana | | UMIC | | Yes | | 2011 | |  | |
| Haiti | | LIC | | No | |  | | HPV vaccine demonstration projects rolled-out in 2009 - 2010 and 2018 | |
| Honduras | | LMIC | | Yes | | 2016 | |  | |
| Jamaica | | UMIC | | Yes | | 2017 | | Introduced in October 2017 | |
| Mexico | | UMIC | | Yes | | 2012 | |  | |
| Netherlands Antilles | |  | |  | |  | | No data found on HPV immunization program | |
| Nicaragua | | LMIC | | No | |  | | No nationwide HPV immunization program | |
| Panama | | HIC | | Yes | | 2008 | |  | |
| Paraguay | | UMIC | | Yes | | 2013 | |  | |
| Peru | | UMIC | | Yes | | 2011 | |  | |
| Puerto Rico | | HIC | | Yes | | 2006 | |  | |
| Sint Maarten (Dutch part) | | HIC | | Yes | | 2013 | |  | |
| St. Kitts and Nevis | | HIC | | No | |  | | No nationwide HPV immunization program. Introduction announced for 2018 | |
| St. Lucia | | UMIC | | No | |  | | No nationwide HPV immunization program | |
| St. Martin (French part) | | HIC | |  | |  | |  | |
| St. Vincent and the Grenadines | | UMIC | | No | |  | | No nationwide HPV immunization program | |
| Suriname | | UMIC | | Yes | | 2013 | |  | |
| Trinidad and Tobago | | HIC | | Yes | | 2013 | |  | |
| Turks and Caicos Islands | | HIC | |  | |  | | No data found on HPV immunization program | |
| Uruguay | | HIC | | Yes | | 2013 | |  | |
| Venezuela, RB | | UMIC | | No | |  | | No nationwide HPV immunization program. Included in national immunization policy in 2015 but remains available through the NIP | |
| Virgin Islands (U.S.) | | HIC | | Yes | | 2006 | |  | |
| **Middle East and North Africa** | | | | | | | | | |
| Algeria | | | UMIC | | No |  | | No nationwide HPV immunization program | |
| Bahrain | | | HIC | | No |  | | No nationwide HPV immunization program | |
| Djibouti | | | LMIC | | No |  | | No nationwide HPV immunization program | |
| Egypt, Arab Republic | | | LMIC | | No |  | | No nationwide HPV immunization program | |
| Iran, Islamic Rep. | | | UMIC | | No |  | | No nationwide HPV immunization program | |
| Iraq | | | UMIC | | No |  | | No nationwide HPV immunization program | |
| Israel | | | HIC | | Yes | 2013 | |  | |
| Jordan | | | UMIC | | No |  | | No nationwide HPV immunization program | |
| Kuwait | | | HIC | | No |  | | No nationwide HPV immunization program | |
| Gaza Strip | | | LMIC | |  |  | | No data found on HPV immunization program | |
| Lebanon | | | UMIC | | No |  | | No nationwide HPV immunization program | |
| Libya | | | UMIC | | Yes | 2013 | |  | |
| Malta | | | HIC | | Yes | 2012 | |  | |
| Morocco | | | LMIC | | No |  | | No nationwide HPV immunization program | |
| Oman | | | HIC | | No |  | | No nationwide HPV immunization program | |
| Qatar | | | HIC | | Yes | 2018 | | Introduced in April 2018 | |
| Saudi Arabia | | | HIC | | No |  | | No nationwide HPV immunization program | |
| Syrian Arab Republic | | | LIC | | No |  | | No nationwide HPV immunization program | |
| Tunisia | | | LMIC | | No |  | | No nationwide HPV immunization program | |
| United Arab Emirates | | | HIC | | Yes | 2008 | |  | |
| West Bank | | | LMIC | | No |  | | No nationwide HPV immunization program | |
| Western Sahara | | |  | |  |  | | No data found on HPV immunization program | |
| Yemen, Rep. | | | LIC | | No |  | | No nationwide HPV immunization program | |
| **North America** | | | | | | | | | |
| Bermuda | HIC | | | | Yes | 2016 | |  | |
| Canada | HIC | | | | Yes | 2007 | |  | |
| Guadeloupe |  | | | |  |  | | No data found on HPV immunization program | |
| Martinique |  | | | |  |  | | No data found on HPV immunization program | |
| Montserrat |  | | | |  |  | | No data found on HPV immunization program | |
| St Pierre & Miquelon |  | | | |  |  | | No data found on HPV immunization program | |
| United States | HIC | | | | Yes | 2006 | |  | |
| **South East Asia** | | | | | | | | | |
| Afghanistan | LIC | | | | No |  | | No nationwide HPV immunization program | |
| Bangladesh | LMIC | | | | No |  | | HPV vaccine demonstration project launched in 2016 for 2 years | |
| Bhutan | LMIC | | | | Yes | 2010 | |  | |
| India | LMIC | | | | No |  | | HPV vaccine demonstration project launched in 2009 | |
| Maldives | UMIC | | | | No |  | | No nationwide HPV immunization program | |
| Nepal | LIC | | | | No |  | | HPV vaccine demonstration program was launched | |
| Pakistan | LMIC | | | | No |  | | No nationwide HPV immunization program | |
| Sri Lanka | LMIC | | | | Yes | 2017 | | Introduced in July 2017 | |
| **Sub-Saharan Africa** | | | | | | | | | |
| Angola | LMIC | | | | Yes | 2015 | | Announced for nationwide introduction in 2015 | |
| Benin | LIC | | | | No |  | | HPV vaccine demonstration project was launched | |
| Botswana | UMIC | | | | Yes | 2015 | |  | |
| Burkina Faso | LIC | | | | No |  | | HPV vaccine demonstration project launched in 2015 | |
| Burundi | LIC | | | | No |  | | HPV vaccine demonstration project was launched | |
| Cameroon | LMIC | | | | No |  | | HPV vaccine demonstration project launched between March 2010 - November 2012 | |
| Cape Verde | LMIC | | | | No |  | | No nationwide HPV immunization program | |
| Central African Republic | LIC | | | | No |  | | No nationwide HPV immunization program | |
| Chad | LIC | | | | No |  | | No nationwide HPV immunization program | |
| Comoros | LIC | | | | No |  | | No nationwide HPV immunization program | |
| Congo, Democratic Republic | LIC | | | | No |  | | No nationwide HPV immunization program | |
| Congo Republic | LMIC | | | | No |  | | No nationwide HPV immunization program | |
| Côte d'Ivoire | LMIC | | | | No |  | | HPV vaccine demonstration project launched in 2015 | |
| Equatorial Guinea | UMIC | | | | No |  | | No nationwide HPV immunization program | |
| Eritrea | LIC | | | | No |  | | No nationwide HPV immunization program | |
| Ethiopia | LIC | | | | Yes | 2018 | |  | |
| Gabon | UMIC | | | | No |  | | No nationwide HPV immunization program | |
| Gambia, The | LIC | | | | No |  | | HPV vaccine demonstration project launched in November 2014 | |
| Ghana | LMIC | | | | No |  | | HPV vaccine demonstration project launched in November 2013 for 2 years | |
| Guinea | LIC | | | | No |  | | No nationwide HPV immunization program | |
| Guinea-Bissau | LIC | | | | No |  | | No nationwide HPV immunization program | |
| Kenya | LMIC | | | | Yes | 2019 | |  | |
| Lesotho | LMIC | | | | Yes | 2012 | |  | |
| Liberia | LIC | | | | Yes | 2019 | |  | |
| Madagascar | LIC | | | | No |  | | HPV vaccine demonstration project rolled-out between 2013 to 2015 | |
| Malawi | LIC | | | | No |  | | HPV vaccine demonstration project rolled-out between 2013 to 2016. Nationwide introduction announced for January 2019 | |
| Mali | LIC | | | | No |  | | HPV vaccine demonstration project was launched | |
| Mauritania | LMIC | | | | No |  | | HPV vaccine demonstration project was launched | |
| Mauritius | UMIC | | | | Yes | 2016 | | Announced in August 2016 | |
| Mayotte |  | | | |  |  | | No data found on HPV immunization program | |
| Mozambique | LIC | | | | No |  | | HPV vaccine demonstration project launched in 2014 | |
| Namibia | UMIC | | | | No |  | | No nationwide HPV immunization program | |
| Niger | LIC | | | | No |  | | HPV vaccine demonstration project rolled-out between 2013 to 2015 | |
| Nigeria | LMIC | | | | No |  | | HPV vaccine demonstration project was launched. Nationwide introduction planned for 2019 | |
| Réunion |  | | | |  |  | | No data found on HPV immunization program | |
| Rwanda | LIC | | | | Yes | 2011 | |  | |
| Saint Helena |  | | | |  |  | | No data found on HPV immunization program | |
| São Tomé and Principe | LMIC | | | | Yes | 2016 | |  | |
| Senegal | LIC | | | | Yes | 2016 | |  | |
| Seychelles | HIC | | | | Yes | 2014 | |  | |
| Sierra Leone | LIC | | | | No |  | | HPV vaccine demonstration project launched in 2012 for 2 years | |
| Somalia | LIC | | | | No |  | | No nationwide HPV immunization program | |
| South Africa | UMIC | | | | Yes | 2014 | |  | |
| South Sudan | LIC | | | | No |  | | No nationwide HPV immunization program | |
| Sudan | LMIC | | | | No |  | | No nationwide HPV immunization program | |
| Swaziland | LMIC | | | | No |  | | No nationwide HPV immunization program | |
| Tanzania | LIC | | | | Yes | 2018 | |  | |
| Togo | LIC | | | | No |  | | HPV vaccine demonstration project was launched | |
| Uganda | LIC | | | | Yes | 2012 | |  | |
| Zambia | LMIC | | | | Yes | 2019 | |  | |
| Zimbabwe | LIC | | | | Yes | 2018 | | Introduced in May 2018 | |

Drawing on data from <http://www.hpvcentre.net>, WHO 2019; Gallagher et al. 2018; LaMontagne et al. 2017; Herrero et al. 2015.

Notes: NIP = National Immunization Program; LIC = Low-Income Country; LMIC = Lower Middle-Income Country; UMIC = Upper Middle-Income Country; HIC = High-income Country.

Country income ranking as defined by the World Bank (<http://data.worldbank.org/about/country-and-lending-groups>). Where the income ranking is not indicated, the information could not be confirmed.

Where nationwide HPV immunization programs have not been identified, it may be that the vaccine is available in the private health sector.

Supplementary Table 2: Literature Search Strategy

| **Query #** | **Search Term** |
| --- | --- |
| #1 | “Immuni* Program” |
| #2 | “National Immuni* Program” OR NIP OR “Expanded Program on Immuni*” OR EPI OR “Universal Immuni* Program” OR UIP OR Immuni* OR “Vaccination” OR “Vaccine Administration” OR Vaccine OR “Vaccine/Introduction” OR “Immuni*/Pilot*” OR “Immuni*/Demonstration Project” OR “Immuni* Program/Communication” OR “Immuni* Program/Community and Partnership” OR “Immuni* Program/Economics” OR “Immuni* Program/Finance” OR “Immuni* Program/Instrumentation” OR “Immuni* Program/Legislation” OR “Immuni* Program/Workforce” OR “Immuni* Program/Organization and Administration” OR “Immuni* Program/Management” OR “Immuni* Program/Supply and Distribution” OR “Immuni* Program/Performance” OR “Immuni* Program/Strengthen*” |
| #3 | #1 OR #2 |
| #4 | “Health System” |
| #5 | “Global Health” OR “Health Service” OR Hospital OR Clinic OR “School Health” OR “Adolescent Health” OR “Health Policy” OR “Health Governance” OR “Health Workforce” OR “Health Worker” OR “Health Care Worker” OR “Health Provider” OR Nurse OR “Medical Products” OR “Medical Technologies” OR “Service Delivery” OR “Health Care” OR “Health Care Delivery” OR “Health Information” OR “Health Financing” OR “Health System/Constraints” OR “Health System/Limitations” OR “Health System/Restrictions” OR “Health System/Barriers” OR “Health System/Challenges” OR “Health System/Facilitators” OR “Health System/Enablers” OR “Health System/Drivers” OR “Health System/Building Blocks” OR “Health System/Strengthen*” OR “Health System/Function*” OR “Health System/Capacity” |
| #6 | #4 OR #5 |
| #7 | #3 AND #6 |
| #8 | “Sub-Saharan Africa” OR “West Africa” OR “East Africa” OR “Central Africa” OR “Southern Africa” |
| #9 | Algeria OR Angola OR Benin OR Botswana OR “Burkina Faso” OR Burundi OR Cameroon OR “Cape Verde” OR “Cabo Verde” OR “Central African Republic” OR Chad OR Comoros OR Comores OR Comoro OR Congo OR “Congo-Brazzaville” OR “Congo Republic” OR “Republic of the Congo” “Côte d'Ivoire” OR “Democratic Republic of the Congo” OR “DR Congo” OR DRC OR “Congo-Kinshasa” OR Djibouti OR “Equatorial Guinea” OR Eritrea OR Ethiopia OR Gabon OR Gambia OR “The Gambia” OR Ghana OR Guinea OR Guinea-Bissau OR Kenya OR Lesotho OR Liberia OR Madagascar OR Malawi OR Mali OR Mauritania OR Mauritius OR Mozambique OR Namibia OR Niger OR Nigeria OR Rwanda OR “Sao Tome and Principe” OR “São Tomé and Príncipe” OR Senegal OR Seychelles OR “Sierra Leone” OR Somalia OR “South Africa” OR “South Sudan” OR Sudan OR Swaziland OR Togo OR Uganda OR “United Republic of Tanzania” OR Tanzania OR Zambia OR Zimbabwe |
| #10 | #8 OR #9 |
| #11 | “Human Papillomavirus/Vaccine” OR “HPV/Vaccine” OR “Cervical Cancer/Vaccine” OR Gardasil OR Cervarix OR “Human Papillomavirus/Vaccination” “Human Papillomavirus/Immuni*” OR “HPV Vaccination” OR “HPV/Immuni*” |
| #12 | #7 AND #10 AND #11 |

Supplementary Table 3: Summary of the quality appraisal of studies included in the systematic review

| **No.** | **Author, year** | **Aim** | **Study design** | **Clear aim^1^** | **Appropriate study design^2^** | **Appropriate methodology^3^** | **Appropriate ethics^4^** | **Appropriate rigour^5^** | **Appropriate analysis^6^** | **Clear findings^7^** | **Reviewers’ comments** |
| --- | --- | --- | --- | --- | --- | --- | --- | --- | --- | --- | --- |
| 1 | Audu et al, 2015 | To document the knowledge of healthcare professionals on HPV vaccine and its acceptability | Cross-sectional, questionnaire-based | Yes | Yes | Yes | Yes | Yes | Yes | Yes | High quality |
| 2 | Ayissi et al, 2012 | To assess awareness, knowledge and beliefs about HPV, cervical cancer HPV vaccine and willingness to vaccinate among adolescent females | Cross-sectional, questionnaire-based | Yes | Yes | Yes | Yes | Yes | Yes | Yes | High quality |
| 3 | Bardají et al, 2018 | To assess the awareness of cervical cancer and HPV infection, and anticipated acceptance of HPV vaccination | Quantitative, cross-sectional | Yes | No | Yes | Yes | No | No | Yes | Low quality. The quantitative study design is not appropriate for the aim. The methods reported are mainly qualitative |
| 4 | Botha et al, 2015 | To investigate acceptance of school-based HPV vaccination | Quantitative | Yes | Yes | Yes | Yes | Yes | Yes | Yes | High quality |
| 5 | Botwright et al, 2017 | To determine the costs of HPV vaccine delivered during Gavi demonstration projects in 12 countries | Quantitative | Yes | Yes | Yes | Yes | Yes | Yes | Yes | High quality |
| 6 | Chigbu et al, 2017 | To determine the impact of trained community health educators on the uptake of cervical and breast cancer screening, and HPV vaccination | Prospective population-based intervention | Yes | Yes | Yes | Yes | Yes | Yes | Yes | High quality |
| 7 | Coleman et al, 2011 | To investigate the acceptability of the HPV vaccine in Ghana | Qualitative, questionnaire-based | Yes | Yes | Yes | Yes | Yes | Yes | Yes | High quality |
| 8 | De Groot et al, 2017 | To assess the knowledge, attitudes, and practices regarding cervical cancer and HPV among teenagers and adults in Mali | Qualitative, household survey | Yes | Yes | Yes | Yes | Yes | Yes | Yes | High quality |
| 9 | DiAngi et al, 2011 | To examine HPV vaccine acceptability for adolescent girls and its predictors among healthcare-seeking adults in Gaborone | Cross-sectional, survey | Yes | Yes | Yes | Yes | Yes | Yes | Yes | High quality |
| 10 | Francis et al, 2011 | To examine women’s attitudes, beliefs and knowledge of HPV and cervical cancer, HPV vaccine awareness and acceptance | Qualitative | Yes | Yes | Yes | Yes | Yes | Yes | Yes | High quality |
| 11 | Francis et al, 2010 | To examine women’s attitudes, knowledge, and beliefs about HPV, cervical cancer, and the HPV vaccine | Quantitative | Yes | Yes | Yes | Yes | Yes | Yes | Yes | High quality |
| 12 | Friedman et al, 2014 | To explore sociocultural factors associated with HPV vaccine acceptability | Qualitative | Yes | Yes | Yes | Yes | Yes | Yes | Yes | High quality |
| 13 | Harries et al, 2009 | To investigate key challenges and barriers towards HPV vaccine introduction in the Western Cape Province, South Africa | Qualitative | Yes | Yes | Yes | Yes | Yes | Yes | Yes | High quality |
| 14 | Hoque, 2015 | To determine the awareness and acceptability of HPV vaccination among university academics | Qualitative, cross-sectional | Yes | Yes | Yes | Yes | Yes | Yes | Yes | High quality |
| 15 | Hoque, 2016 | To investigate factors contributing to recommendation of HPV vaccines to patients | Quantitative, cross-sectional | Yes | Yes | Yes | Yes | Yes | Yes | Yes | High quality |
| 16 | Hoque et al, 2013 | To assess the awareness of cervical cancer and its risk factors among female undergraduates and to determine the level of acceptability of HPV vaccination | Qualitative, cross-sectional | Yes | Yes | Yes | Yes | Yes | Yes | Yes | High quality |
| 17 | Hutubessy et al, 2012 | To cost and plan the roll-out of HPV vaccines nationwide as part of the national comprehensive cervical cancer prevention and control strategy | Quantitative | Yes | Yes | Yes | Yes | Yes | Yes | Yes | High quality |
| 18 | Kamya et al, 2017 | To understand how a partnership around HPV vaccine, which targets adolescent girls to prevent cervical cancer, might involve a different group of stakeholders than traditional childhood vaccines | Mixed-methods case study | Yes | Yes | Yes | Yes | Yes | Yes | Yes | High quality |
| 19 | Katz et al, 2013 | To elucidate factors influencing HPV vaccination among a sample of low-income South African adolescents receiving the vaccine for the first time in Soweto | Qualitative | Yes | Yes | Yes | Yes | Yes | Yes | Yes | High quality |
| 20 | Ladner et al, 2012 | To describe the results of and key concerns in eight HPV vaccination programs conducted in seven lowest income countries through the Gardasil Access Program (GAP) | Mixed-methods | Yes | Yes | Yes | Yes | Yes | Yes | Yes | High quality |
| 21 | Ladner et al, 2014 | To analyse HPV vaccination programs performance implemented in LMICs | Quantitative | Yes | Yes | Yes | Yes | Yes | Yes | Yes | High quality |
| 22 | LaMontagne et al, 2011 | To assess HPV vaccination coverage after demonstration projects conducted in India, Peru, Uganda and Vietnam by PATH and national governments and to explore the reasons for vaccine acceptance or refusal | Mixed-methods, cross-sectional | Yes | Yes | Yes | Yes | Yes | Yes | Yes | High quality |
| 23 | Levin et al, 2013 | To estimate the incremental delivery cost of HPV vaccination of young adolescent girls in Peru, Uganda and Vietnam | Mixed-method | Yes | Yes | Yes | Yes | Yes | Yes | Yes | High quality |
| 24 | Mabeya et al, 2018 | To identify barriers and facilitators associated with uptake of HPV vaccine | Cross-sectional, questionnaire-based | Yes | Yes | Yes | Yes | Yes | Yes | Yes | High quality |
| 25 | MacPhail et al, 2013 | To assess the acceptability and feasibility of introducing HPV vaccination in the South African public health sector, and particularly the notion of using the vaccination schedule of three repeat visits as the basis for providing adolescents with additional health service | Qualitative, cross-sectional | Yes | Yes | Yes | Yes | Yes | Yes | Yes | High quality |
| 26 | Makwe and Anorlu, 2011 | To assess awareness and knowledge of HPV infection and vaccines and to assess attitude toward these vaccines among female nurses | Qualitative, cross-sectional | Yes | Yes | Yes | Yes | Yes | Yes | Yes | High quality |
| 27 | Masika et al, 2015 | To assess primary school teachers’ knowledge and acceptability of HPV vaccine and to explore facilitators and barriers of an ongoing Gavi Alliance-supported vaccination program | Mixed-methods, cross-sectional, | Yes | Yes | Yes | Yes | Yes | Yes | Yes | High quality |
| 28 | Massey et al, 2017 | To examine HPV vaccine awareness and receptivity among adolescents and young adults in Senegal | Quantitative, questionnaire-based | Yes | Yes | Yes | Yes | Yes | Yes | Yes | High quality |
| 29 | Moodley et al, 2013 | To demonstrate the capacity of school health teams to carry out vaccinations within a school environment | Mixed-methods | Yes | Yes | Yes | Yes | Yes | Yes | Yes | High quality |
| 30 | Morhason-Bello et al, 2015 | To determine the willingness of reproductive-aged women in a Nigerian community to allow HPV vaccination for their children and the associated factors with this decision | Quantitative, multi-stage household survey | Yes | Yes | Yes | Yes | Yes | Yes | Yes | High quality |
| 31 | Msyamboza et al, 2017 | To evaluate HPV vaccine coverage, lessons learnt, and challenges identified during the first three years of implementation | Mixed-method, cross-sectional | Yes | Yes | Yes | Yes | Yes | Yes | Yes | High quality |
| 32 | Mugisha et al, 2015 | To explore the feasibility of two HPV vaccine delivery strategies; school-based and age-based | Qualitative | Yes | Yes | Yes | Yes | Yes | Yes | Yes | High quality |
| 33 | Ndizeye et al, 2018 | To document the knowledge and practices of GPs at district hospital level towards invasive cervical cancer control | Descriptive, cross-sectional | Yes | Yes | Yes | Yes | Yes | Yes | Yes | High quality |
| 34 | Ngabo et al, 2015 | To estimate the cost of introducing and delivering each of the three vaccines nationwide in Rwanda | Quantitative | Yes | Yes | Yes | Yes | Yes | Yes | Yes | High quality |
| 35 | Odunyemi et al, 2018 | To evaluate the effect of nursing intervention on mothers’ knowledge of cervical cancer and acceptance of HPV vaccination for their adolescent daughters | Quasi-experimental study | Yes | Yes | Yes | Yes | Yes | Yes | Yes | High quality |
| 36 | Ogembo et al, 2014 | To inform the Cameroon MOH of the acceptability, feasibility, and optimal delivery strategies for HPV vaccine | Project evaluation | Yes | Yes | Yes | Yes | Yes | Yes | Yes | High quality |
| 37 | Okunade et al, 2017 | To determine the knowledge and acceptability of HPV vaccine among women | Descriptive, cross-sectional | Yes | Yes | Yes | Yes | Yes | Yes | Yes | High quality |
| 38 | Poole et al, 2013 | To determine HPV knowledge, acceptability, and other factors associated with the feasibility of HPV vaccine implementation in Mali | Qualitative, cross-sectional | Yes | Yes | Yes | Yes | Yes | Yes | Yes | High quality |
| 39 | Ports et al, 2013 | To elucidate potential barriers and facilitators to HPV vaccination in Malawi | Qualitative | Yes | Yes | Yes | Yes | Yes | Yes | Yes | High quality |
| 40 | Quentin et al, 2012 | To estimate thecosts of a school-based HPV vaccination project in three districts in Mwanza Region | Mixed-methods | Yes | Yes | Yes | Yes | Yes | Yes | Yes | High quality |
| 41 | Remes et al, 2012 | To learn what people knew about cervical cancer and HPV vaccination, whether they would find HPV vaccination acceptable | Qualitative | Yes | Yes | Yes | Yes | Yes | Yes | Yes | High quality |
| 42 | Tchounga et al 2012 | To assess knowledge, attitudes and practices of midwives towards cervical cancer prevention | Qualitative, cross-sectional | Yes | Yes | Yes | Yes | Yes | Yes | Yes | High quality |
| 43 | Torres-Rueda et al, 2016 | To evaluate the impact of the HPV vaccine introduction on the country’s immunisation programme and health system | Mixed-methods | Yes | Yes | Yes | Yes | Yes | Yes | Yes | High quality |
| 44 | Tuhiro et al, 2014 | To assess girls’ knowledge of cervical cancer and HPV vaccine, and their acceptance of future vaccination of friends and hypothetical daughters | Cross-sectional, mixed-methods | Yes | Yes | Yes | Yes | Yes | Yes | Yes | High quality |
| 45 | Tuhiro et al, 2017 | To explore community member’s perceptions about HPV vaccination in Ibanda district and the implications of the perceptions for acceptability of HPV vaccination | Qualitative | Yes | Yes | Yes | Yes | Yes | Yes | Yes | High quality |
| 46 | Ugwu et al, 2013 | To determine the awareness and acceptability of the HPV vaccine and screening for cervical cancer among female health-care workers | Cross-sectional, questionnaire-based | Yes | Yes | Yes | Yes | Yes | Yes | Yes | High quality |
| 47 | Umeh et al, 2016 | To assess Nigerian mothers’ willingness-to-pay (WTP) for HPV vaccine | Cross-sectional, survey | Yes | Yes | Yes | Yes | Yes | Yes | Yes | High quality |
| 48 | Urasa and Darj, 2011 | To determine nurses’ awareness of cervical cancer and their own screening practices at a hospital in Tanzania | Descriptive, cross-sectional | Yes | Yes | Yes | Yes | No | Yes | Yes | Moderate quality. The limitations of the study are not adequately addressed |
| 49 | Venturas and Umeh, 2017 | To explore health professionals’ perspectives on the HPV vaccination programme in Zambia | Qualitative | Yes | Yes | Yes | Yes | Yes | Yes | Yes | High quality |
| 50 | Vermandere et al, 2015 | To evaluate the implementation of the HPV vaccination demonstration program in Eldoret | Qualitative | Yes | Yes | Yes | Yes | Yes | Yes | Yes | High quality |
| 51 | Vermandere et al, 2014 | To survey the acceptability, subsequent uptake and encountered barriers from the perspective of the mothers of young girls, in the context of a pilot HPV vaccination program | Qualitative, longitudinal study | Yes | Yes | Yes | Yes | Yes | Yes | Yes | High quality |
| 52 | Wamai et al, 2013 | To investigate the knowledge and awareness of HPV, primary cause of cervical cancer and HPV vaccine among nurses | Qualitative, questionnaire-based | Yes | Yes | Yes | Yes | Yes | Yes | Yes | High quality |
| 53 | Watson-Jones et al, 2015 | To determine facilitators and barriers to HPV vaccination and potential acceptability of a future HPV vaccination programme amongst girls living in hard-to-reach populations | Qualitative | Yes | Yes | Yes | Yes | Yes | Yes | Yes | High quality |
| 54 | Watson-Jones et al, 2012 | To examine the characteristics of receivers and non-receivers of HPV vaccination in Tanzania and identified reasons for not receiving the vaccine | Qualitative | Yes | Yes | Yes | Yes | Yes | Yes | Yes | High quality |

Notes:

^1^Is the aim clearly stated?

^2^Is the study design appropriate to address the aim?

^3^Is the methodology sound and appropriate for addressing the aim?

^4^have ethical considerations been sufficiently addressed?

^5^Is there clear consideration for reliability/bias/reflexivity/limitations/rigour

^6^Is the data analysis appropriate for the aim?

^7^Are the findings clearly described?

Supplementary Table 4: Data Extraction Table

| **Study No.** | **Study Details** | | | **Context** | | **Data Extraction** | | | | | |
| --- | --- | --- | --- | --- | --- | --- | --- | --- | --- | --- | --- |
|  | Author and Year | Title | Country | Underlying Context | Availability / Type of HPV Immunization Program | Main Theme | Health Systems Constraint | Health Systems Facilitator | Comment 1 | Comment 2 | Comment 3 |
| 1 | Audu et al. 2015 | Awareness and perception of human papilloma virus vaccine among healthcare professionals in Nigeria | Nigeria | Not Applicable | Not Available | Health Workers | Inadequate training of health workers |  | 91.0% were aware of HPV while 44.0% knew about the HPV vaccine. 81.0% would allow their teenage daughters to be vaccinated | Reasons given for refusing HPV vaccination: lack of knowledge about the safety of the vaccine, immorality, doubt about vaccine potency | Nurses and midwives were the least aware about the HPV vaccine (p = 0.001) |
| 2 | Ayissi et al. 2012 | Awareness, acceptability and uptake of human papilloma virus vaccine among Cameroonian school-attending female adolescents | Cameroon | Socio-demographic | Demonstration Project | Information / Community Engagement |  | Appropriate communication and sensitization strategies | 34.2% of the respondents had been vaccinated against HPV infection | 75.9% were aware that that HPV vaccine is important in preventing HPV infections and cervical cancer | 62.9% heard about HPV from a nurse. There was a high acceptability of HPV vaccination |
| 3 | Bardají et al. 2018 | Awareness of cervical cancer and willingness to be vaccinated against human papillomavirus in Mozambican adolescent girls | Mozambique | Not Applicable | Demonstration Project | Information / Community Engagement |  | Appropriate health education and sensitization about HPV vaccine | Increased acceptability of HPV vaccine among adolescent girls was associated with level of education |  |  |
| 4 | Botha et al. 2015 | The Vaccine and Cervical Cancer Screen (VACCS) project: Acceptance of human papillomavirus vaccination in a school-based programme in two provinces of South Africa | South Africa | Socio-economic | Demonstration Project | Information / Community Engagement |  | Appropriate communication and sensitization strategies | High acceptance rate following better information underlines the importance of clear, direct communication with parents | Parents who received complete information on the HPV vaccine demonstrated a very high acceptance rate | Appropriate information contributed significantly to vaccine uptake |

| 5 | Botwright et al. 2017 | Experiences of operational costs of HPV vaccine delivery strategies in Gavi-supported demonstration projects | African Countries | Socio-economic | Demonstration Project | Health Financing | Non-integration of HPV immunization programs within existing routine immunization programs | Planning appropriate delivery strategies based on local context. Evidence-based social mobilization | Social mobilization, information, education, and communication (IEC) and service delivery each comprised the greatest financial cost | Countries incurring the greatest financial costs also had the highest social mobilization/IEC costs | School-based delivery had the highest service delivery cost per dose due to transportation costs and health worker per diems |
| --- | --- | --- | --- | --- | --- | --- | --- | --- | --- | --- | --- |
| 6 | Chigbu et al. 2017 | The impact of community health educators on uptake of cervical and breast cancer prevention services in Nigeria | Nigeria | Socio-economic | Demonstration Project | Health Workers |  | Educating and training nurses on the HPV vaccination schedule and technique | The intervention was house-to-house cervical and breast cancer prevention education by trained health workers | At baseline, only 2 (0.9%) children had received HPV vaccination. This increased to 33.2% after the intervention (p<0.001) | Education of mothers improves the uptake of HPV vaccination of children |
| 7 | Coleman et al. 2011 | HPV vaccine acceptability in Ghana, West Africa | Ghana | Socio-economic | Available in Private Sector Only | Information / Community Engagement | Lack of awareness, poor knowledge about cervical cancer and HPV vaccine | Education, social mobilization, communication about HPV and the vaccine | Although majority of the women had heard about cervical cancer (87%) and Pap test (63%) before the survey, fewer women had heard about the HPV vaccine (40%) | Participants proved very accepting of the HPV vaccine, both for themselves and their daughters (94% each) | Those less likely to accept the vaccine had significantly lower level of income |
| 8 | De Groot et al. 2017 | Knowledge, attitudes, practices and willingness to vaccinate in preparation for the introduction of HPV vaccines in Bamako, Mali | Mali | Socio-economic | Demonstration Project | Information / Community Engagement |  | Education and communication about HPV, cervical cancer and HPV vaccination | Before the educational session, awareness about HPV was low; 8.6% knew that HPV was transmitted by sexual contact | Knowledge of the participants significantly increased after the education session. | The education session increased the HPV vaccine acceptance in all groups, especially among adolescents |
| 9 | DiAngi et al. 2011 | A cross-sectional study of HPV vaccine acceptability in Gaborone, Botswana | Botswana | Socio-economic | Available in Private Sector Only | Information / Community Engagement |  | Education and communication about HPV, cervical cancer and HPV vaccination | Prior to the survey, few (9%) respondents had heard of HPV vaccine, and most (75%) wanted more information about it | 80% percent of respondents said they would obtain the HPV vaccine for their adolescent daughters |  |
| 10 | Francis et al. 2011 | A qualitative analysis of South African women's knowledge, attitudes, and beliefs about HPV and cervical cancer prevention, vaccine awareness and acceptance, and maternal-child communication about sexual health | South Africa | Socio-economic | Available in Private Sector Only | Information / Community Engagement | Poor access to information about HPV, cervical cancer and HPV vaccine |  | Majority of participants knew little about HPV, cervical cancer, and the HPV vaccine, and they expressed interest in learning more about these topics | Participants agreed that vaccinations would keep their children healthy, but they worried about long-term side effects | Most participants thought the government should offer the vaccine for free as part of the country’s immunization program |
| 11 | Francis et al. 2010 | Examining attitudes and knowledge about HPV and cervical cancer risk among female clinic attendees in Johannesburg, South Africa | South Africa | Socio-economic | Not Available | Information / Community Engagement | Poor access to information about HPV, cervical cancer and HPV vaccine |  | 39% of participants had not heard of cervical cancer while 71% had never heard of HPV | Almost half of participants were very likely to vaccinate if their health provider recommended it | Commonly cited barriers to vaccinating included inadequate information, fear of side effects, cost of vaccine. |
| 12 | Friedman et al. 2014 | Preparing for human papillomavirus vaccine introduction in Kenya: implications from focus-group and interview discussions with caregivers and opinion leaders in Western Kenya | Kenya | Socio-cultural | Not Available | Information / Community Engagement | Poor access to information about HPV, cervical cancer and HPV vaccine | Effective Community sensitization about HPV vaccination through culturally appropriate strategies | Majority of caregivers and opinion leaders had not previously heard of cervical cancer | Apart from health and media experts (n = 3), none of the participants had heard of the HPV vaccine | Participants identified cultural beliefs as potential barriers to vaccination |
| 13 | Harries et al. 2009 | Preparing for HPV vaccination in South Africa: key challenges and opinions | South Africa | Not Applicable | Available in Private Sector Only | Service Delivery | Health resource constraints. Logistical challenges with vaccine delivery | Appropriate health promotion strategies. Partnerships between Departments of Education + Health, private + public health sectors | Policy influencers and health providers expressed strong support for the HPV vaccine | Participants acknowledged the difficulties of introducing a new technology in a health system with limited capacity and resources - especially where school health is concerned | Opposition could be countered by providing comprehensive information and communication with all stakeholders about the benefits of the HPV immunization |
| 14 | Hoque 2015 | Acceptability of human papillomavirus vaccination among academics at the University of KwaZulu-Natal, South Africa | South Africa | Socio-economic | Available through NIP | Information / Community Engagement | Poor knowledge about safety and effectiveness of the vaccine | Adequate knowledge about HPV, cervical cancer and HPV vaccine | Almost all (96%) the academics had heard of cervical cancer and all had heard about HPV | After reading information on cervical cancer and HPV, the vaccine acceptability rate increased from 80% to 89% | Those who still did not want to vaccinate their daughters cited the “newness” of the vaccine and fear of side-effects. |
| 15 | Hoque 2016 | Factors influencing the recommendation of the Human Papillomavirus vaccine by South African doctors working in a tertiary hospital | South Africa | Not Applicable | Available through NIP | Health Workers | Low level of knowledge on HPV infection and vaccination among Doctors |  | 75.3% of Doctors indicated not knowing the effectiveness of the HPV vaccine | Only one doctor (of 320) agreed that the vaccines should be given to girls before sexual debut | 96.3% of the Doctors indicated willingness to recommend the vaccines to their patients |
| 16 | Hoque et al 2013 | Human Papillomavirus vaccination acceptability among female university students in South Africa | South Africa | Not Applicable | Available in Private Sector Only | Information / Community Engagement | Low level of knowledge on HPV infection, cervical cancer, and HPV vaccine |  | Overall, awareness of cervical cancer and HPV was low | Of the 163 students who reported never having had sex, 127 (77.3%) were willing to accept HPV vaccination | Reasons for non-acceptance, included fear of injection (78.9%), side-effects (15.8%), and pain (5.3%) |
| 17 | Hutubessy et al. 2012 | A case study using the United Republic of Tanzania: Costing nationwide HPV vaccine delivery using the WHO Cervical Cancer Prevention and Control Costing Tool | Tanzania | Not Applicable | Not Available | Health Financing | Financial costs of social mobilization and HPV vaccine delivery |  | Excluding vaccine procurement, social mobilization and IEC contributed to the largest share of costs, followed by service delivery | Substantial funding is required to facilitate HPV vaccine delivery | Recurrent costs per dose would be higher for a school-based compared to health facility-based delivery because of transportation costs and health worker per diems |
| 18 | Kamya et al. 2017 | Evaluating global health partnerships: A case study of a Gavi HPV vaccine application process in Uganda | Uganda | Political | Available through NIP | Governance / Policy | Weak involvement Ministries of Education and Finance | Clear governance and management structures and processes. Political champions (First Lady). Advocacy by NGOs (PATH) | Respondents agreed that partnerships increased country ownership and improved the effectiveness and efficiency of the GAVI application process | Potential drawbacks of the partnership included an unnecessary management burden on organization | Network mapping identified the absence of the Ministries of education and Finance |
| 19 | Katz et al. 2013 | A qualitative analysis of factors influencing HPV vaccine uptake in Soweto, South Africa among adolescents and their caregivers | South Africa | Socio-economic | Available in Private Sector Only | Health Workers |  | The influence of health workers | Health workers at the adolescent clinic were serves as “adult proxies” and positively influenced HPV vaccine uptake | High HIV endemicity, sexual violence, poverty, and an abundance of female-headed households influenced vaccine uptake |  |
| 20 | Ladner et al. 2012 | Assessment of eight HPV vaccination programs implemented in lowest income countries | Lesotho and Cameroon (non-African countries included) | Socio-economic | Demonstration Project | Service Delivery |  | Use of mixed vaccine delivery models; school and health facility -based strategies | Health-facility model had a lower vaccine coverage (77.1%) compared to the school-based (93.0%) or mixed model (93.8%) (p = 0.74) | With regards to adherence to the vaccine doses, the mixed model was the most effective | In Cameroon, intensive IEC strategies were facilitators to the success of HPV vaccination campaigns |
| 21 | Ladner et al. 2014 | Performance of 21 HPV vaccination programs implemented in low and middle-income countries, 2009-2013 | Cameroon, Kenya, Lesotho, Tanzania and Uganda (non-African LMICs included) | Socio-economic | Demonstration Project | Service Delivery |  | Use of school-based delivery models. Community engagement. Adequate communication, especially on vaccine safety and efficacy | School-based vaccine delivery and program management by an NGO (vs MoH) each had a positive and statistically significant impact on vaccine uptake rates | Community involvement appeared to have a positive impact on the vaccine uptake rate | Inclusion of key messages regarding the safety and efficacy of the vaccine had a positive impact on vaccine uptake |
| 22 | LaMontagne et al. 2011 | Human papillomavirus vaccine delivery strategies that achieved high coverage in low- and middle-income countries | Uganda (non-African LMICs included) | Socio-economic | Demonstration Project | Service Delivery | Use of age-based vaccine delivery models. Low level of awareness about HPV vaccine and vaccination program | Use of grade-based vaccine delivery models Parents' perceived benefits of HPV vaccine due to sensitization and trust in existing NIP | High HPV vaccination coverage was achieved with the grade-based compared to the age-based vaccine delivery strategy in Uganda | The most frequently cited reasons for non-vaccination was a lack of awareness of the immunization programme | Difficulty in determining girl’s eligibility was the mainly challenge with the age-based vaccine delivery model |
| 23 | Levin et al. 2013 | Delivery cost of human papillomavirus vaccination of young adolescent girls in Peru, Uganda and Viet Nam | Uganda (non-African LMICs included) | Socio-economic | Demonstration Project | Health Financing | Cost of delivering vaccines to adolescents is higher than that of routine childhood immunization | Investments in community mobilization and sensitization increases vaccine acceptance and uptake | School-based vaccine delivery incurred a higher economic cost than the health facility-based or integrated outreach models | The average incremental economic cost per fully immunized girl was highest with school-based delivery model | Community mobilization and IEC activities accounted for approximately 40% of start-up costs |
| 24 | Mabeya et al. 2018 | Uptake of three doses of HPV vaccine by primary school girls in Eldoret, Kenya; a prospective cohort study in a malaria endemic setting | Kenya | Disease Epidemiology | Demonstration Project | Service Delivery | Low level of awareness about HPV. Distance to health facility. |  | Of those who received the first dose of HPV vaccine (3026), 63.8% returned for the second dose, and 39.1% for the third dose | Administration of the 2^nd^ dose and caregiver's knowledge of HPV were predictors of uptake of the 3^rd^ dose | Distance to the health facility was a significant barrier to uptake of subsequent doses of the HPV vaccine |
| 25 | MacPhail et al. 2013 | Using HPV vaccination for promotion of an adolescent package of care: Opportunity and perspectives | South Africa | Socio-economic | Available in Private Sector Only | Information / Community Engagement | Poor access to information about HPV, cervical cancer and HPV vaccine |  | Participants expressed demand for information about the HPV vaccine | Community members echoed adolescents’ demands for information about the HPV vaccine | There was an expressed need for integrating other adolescent health programs with HPV immunization |
| 26 | Makwe and Anorlu 2011 | Knowledge of and attitude toward human papillomavirus infection and vaccines among female nurses at a tertiary hospital in Nigeria | Nigeria | Not Applicable | Not Available | Health Workers | Low level of knowledge on HPV, cervical cancer prevention and HPV vaccine |  | 99.4% had heard of cervical cancer and 84.8% had heard of HPV infection. Only 13% knew it could be prevented through vaccination | Most (74.7%) of the respondents had never heard of the HPV vaccines | Reasons for not recommending HPV vaccination included: young age, poor knowledge of risk factors and vaccine safety |
| 27 | Masika et al. 2015 | Knowledge on HPV vaccine and cervical cancer facilitates vaccine acceptability among school teachers in Kitui County, Kenya | Kenya | Socio-economic | Demonstration Project | Information / Community Engagement | Lack of information about HPV and HPV vaccine. poor road networks to schools | Effective social mobilization. Engaging teachers at the outset of the HPV immunization program | 95% of participants knew that the HPV vaccine prevents cervical cancer, but they had very little information about HPV infection and cervical cancer | 89% would allow their daughter or close relative to receive the vaccine. | Vaccine refusal was mainly associated with concerns about vaccine safety |
| 28 | Massey et al. 2017 | Human papillomavirus (HPV) awareness and vaccine receptivity among Senegalese adolescents | Senegal | Socio-economic | Demonstration Project | Information / Community Engagement | Low level of awareness about HPV and HPV vaccine. Poor socio-economic status | Appropriate health worker re-commendation on HPV vaccine | 27% of participants had heard of HPV. Only 28% of those who had heard about HPV indicated willingness to vaccinate | Health providers’ re-commendations were strongly associated with willingness to vaccinate | As socio-economic status increased, the odds of having heard of HPV also increased |
| 29 | Moodley et al. 2013 | High uptake of Gardasil vaccine among 9 – 12-year-old schoolgirls participating in an HPV vaccination demonstration project in KwaZulu-Natal Province, South Africa | South Africa | Not Applicable | Available in Private Sector Only | Service Delivery | Use of paper-based vaccine records | School-based vaccine delivery strategy. Well-trained school health team. Intensive community mobilization and sensitization | 99.7% received their first dose of vaccine, 97.9% received their second dose, and 97.8% received their third dose. | High uptake of the vaccine depends on parental or caregiver consent | Using predominantly paper-based vaccine records was cited as a barrier to real-time monitoring and evaluation of the program |
| 30 | Morhason-Bello et al. 2015 | Willingness of reproductive-aged women in a Nigerian community to accept human papillomavirus vaccination for their children | Nigeria | Not Applicable | Available in Private Sector Only | Information / Community Engagement | Low level of awareness about HPV and HPV vaccine | Free, universal HPV vaccination | 64.3% strongly agreed that HPV vaccination should be made mandatory to all female children | Concerns about the vaccine included: cost (10.2%) and fear of encouraging promiscuity (9.9%), early sexual debut (6.7%), infertility (6.3%), and side-effects (6.0%) | 88.6% women were willing to vaccinate their children |
| 31 | Msyamboza et al. 2017 | Implementation of a human papillomavirus vaccination demonstration project in Malawi: successes and challenges | Malawi | Not Applicable | Demonstration Project | Service Delivery | Use of health facility-based or community outreach delivery models. Low level awareness about the safety of the vaccine | Using a school-based vaccine delivery strategy | 23,831 (89.0%) of school girls and 403 (52.7%) out-of-school girls aged 9–13 years were fully vaccinated | Reasons cited for no or partial vaccination included girls transferring out of the district, dropping out of, and absence from school, and caregivers’ dissent |  |

| 32 | Mugisha et al. 2015 | Feasibility of delivering HPV vaccine to girls aged 10 to 15 years in Uganda | Uganda | Not Applicable | Demonstration Project | Service Delivery | Use of age-based vaccine delivery strategy | Use of grade-based vaccine delivery strategy. Active participation by teachers. Government endorsement of the vaccine | Use of the grade-based strategy achieved higher vaccination coverage compared to the age-based strategy | Establishing eligibility was easier with the grade-based compared to the age-based strategy | The dropout rate between the 1^st^ and 3^rd^ dose was higher using the age-based model |
| --- | --- | --- | --- | --- | --- | --- | --- | --- | --- | --- | --- |
| 33 | Ndizeye et al. 2018 | Knowledge and practices of general practitioners at district hospitals towards cervical cancer prevention in Burundi, 2015: A cross-sectional study | Burundi | Not Applicable | Not Available | Health Workers | Low level of knowledge about HPV vaccination among General Practitioners (GPs) |  | Of 131 GPs, 89% knew that HPV infection is associated with most cervical cancer cases | 55% of GPs were aware that the HPV vaccine can prevent invasive cervical cancer | Only 10.7% knew the target age group for HPV immunization and 3.1% knew the required doses |
| 34 | Ngabo et al. 2017 | A cost comparison of introducing and delivering pneumococcal, rotavirus and human papillomavirus vaccines in Rwanda | Rwanda | Socio-economic | Available through NIP | Health Financing | High financial cost of HPV vaccine delivery due to greater resource requirements |  | The financial cost of delivering the HPV vaccine was significantly higher compared to other routine childhood vaccines | HPV vaccine introduction costs were higher due to greater social mobilization and training requirements | Recurrent costs were greater for the HPV vaccine than for routine childhood vaccines |
| 35 | Odunyemi et al. 2018 | Effect of nursing intervention on mothers' knowledge of cervical cancer and acceptance of human papillomavirus vaccination for their adolescent daughters in Abuja - Nigeria | Nigeria | Not Applicable | Available in Private Sector Only | Health Workers | Lack of education on HPV, cervical cancer and HPV vaccination. Cost of vaccination | Adequate education and communication from nurses about HPV, cervical cancer and HPV vaccination | Prior to the intervention 74% in the experimental arm and 83.1% in the control group had heard about cervical cancer | 85.5% of the respondents cited lack of information as a deterrent to vaccinating their daughters | HPV vaccine acceptance increased after the intervention |
| 36 | Ogembo et al. 2014 | Achieving high uptake of human papillomavirus vaccine in Cameroon: Lessons learned in overcoming challenges | Cameroon | Socio-economic | Demonstration Project | Service Delivery | Negative media reports about the side-effects and interference in HPV immunization program | Use of mixed vaccine delivery models. Adequate community sensitization. Community engagement, especially with municipal and religious leaders | Low vaccine uptake despite adequate social mobilization was linked with false reports by a newspaper article claiming the vaccine had serious side effects including death | The use of mixed vaccine delivery models involving school-based and community outreach approaches was feasible | Wealthy community members subsidized the cost of immunization for those who could not afford to pay |
| 37 | Okunade et al. 2017 | Knowledge and acceptability of human papillomavirus vaccination among women attending the gynaecological outpatient clinics of a university teaching hospital in Lagos, Nigeria | Nigeria | Socio-demographic | Available in Private Sector Only | Information / Community Engagement | High cost of vaccination. Poor knowledge of the safety of the vaccine. Limited access to the HPV vaccine |  | 36.5% of the respondents had heard about genital HPV infection and 18.9% were aware of the existence of HPV vaccines | 81.8% of the respondents expressed their willingness to vaccinate their daughters | Reasons unwillingness to vaccinate were high cost (55.6%) concerns about side-effects (48.1%), and poor availability (25.9%) of the vaccine |
| 38 | Poole et al. 2013 | A cross-sectional study to assess HPV knowledge and HPV vaccine acceptability in Mali | Mali | Socio-cultural | Not Available | Information / Community Engagement | Low level of knowledge about HPV and cervical cancer |  | Participants exhibited low level of knowledge about HPV and cervical cancer | 100% of participants said they would like the HPV vaccine to be available in Mali | There was a high level of willingness to vaccinate |
| 39 | Ports et al. 2013 | Barriers and facilitators to HPV vaccination: Perspectives from Malawian women | Malawi | Socio-cultural | Not Available | Information / Community Engagement | Long distance to health facilities. Inadequate IEC strategies about HPV, cervical cancer and HPV vaccine | Health worker re-commendation on HPV vaccine. Adequate IEC and community engagement | Participants’ knowledge about HPV and cervical cancer was limited. None of the women had heard of the HPV vaccine | Long distance to health facilities negatively influenced women’s ability to access health services and their decision to vaccinate | All participants were accepting of the vaccine once informed about it and were willing to recommend the vaccine to others |
| 40 | Quentin et al. 2012 | Costs of delivering human papillomavirus vaccination to schoolgirls in Mwanza Region, Tanzania | Tanzania | Socio-economic | Demonstration Project | Health Financing | Using an age-based vaccine delivery strategy – less cost effective | Using a class or grade-based vaccine delivery strategy – more cost-effective | Marked difference in the costs of delivering HPV vaccines to girls between age-based and class-based deliver | The cost per fully-immunized schoolgirl using a class-based delivery strategy was lower |  |
| 41 | Remes et al. 2012 | A qualitative study of HPV vaccine acceptability among health workers, teachers, parents, female pupils, and religious leaders in northwest Tanzania | Tanzania | Not Applicable | Not Available | Information / Community Engagement | Low level of awareness about HPV, cervical cancer and HPV vaccine. Fragile health worker capacity and limited health resources | Appropriate community sensitization | Nurses' knowledge was limited. None of the parents, teachers, religious leaders or the girls had heard about HPV or the vaccine. | Most respondents welcomed the vaccine. Almost all parents indicated they would vaccinate their daughters. All the girls wanted to be vaccinated | Health workers were concerned about staff shortages and lack of transport if the HPV immunization program was to be introduced in the country |
| 42 | Tchounga et al. 2012 | Cervical cancer prevention in reproductive health services: Knowledge, attitudes and practices of midwives in Côte d'Ivoire, West Africa | Côte d'Ivoire | Socio-demographic | Available in Private Sector Only | Health Workers | Low level of knowledge about cervical cancer and HPV vaccine | Appropriate training of health workers on cervical cancer and cervical cancer prevention | Only 298 midwives (50.3%) were aware of the HPV vaccine and knew that it protects against cervical cancer | 38.5% of the midwives knew that the vaccine was already available in Côte d’Ivoire | Factors associated with appropriate knowledge were professional experience, training |
| 43 | Torres-Rueda et al. 2016 | HPV vaccine introduction in Rwanda: Impacts on the broader health system | Rwanda | Socio-economic | Available through NIP | Service Delivery |  | Appropriate training, supervision, planning, partnerships, and political will. Strengthening surveillance | HPV vaccine-specific supervision was reported by 10/27 health facilities and were said to have taken place before and during immunizations | Surveillance of adverse events following immunization was emphasised during planning and formed part of training of health workers | A cold chain inventory was carried out before the introduction of the HPV vaccine |
| 44 | Tuhiro et al. 2014 | Effect of school-based human papillomavirus (HPV) vaccination on adolescent girls' knowledge and acceptability of the HPV vaccine in Ibanda District in Uganda | Uganda | Socio-demographic | Demonstration Project | Information / Community Engagement | Mis-information about the side-effects of the HPV vaccine | Pre-immunization sensitization | Vaccinated girls who were sensitized during the demonstration project had a higher level of knowledge about cervical cancer and the HPV vaccine | Motivation for HPV vaccine acceptability was related to appreciation of its preventive role against cervical cancer | Introduction of the HPV vaccine triggered several rumours which threatened acceptability of the vaccine |
| 45 | Tuhiro et al. 2017 | Perceptions of human papillomavirus vaccination of adolescent schoolgirls in western Uganda and their implications for acceptability of HPV vaccination: A qualitative study | Uganda | Socio-cultural | Demonstration Project | Information / Community Engagement | Mis-information about the benefits of the HPV vaccine | Existing perceptions that vaccination in general prevents disease due to the availability of a well-functioning NIP | Following IEC and community mobilization, respondents perceived the HPV vaccine to be beneficial | Misconceptions potentially threatened acceptance; Some believed that the vaccine prevented HIV and pregnancy | Misconceptions about safety of the HPV vaccine persisted despite dissemination of information about vaccine safety |
| 46 | Ugwu et al. 2013 | Acceptability of human papilloma virus vaccine and cervical cancer screening among female health-care workers in Enugu, Southeast Nigeria | Nigeria | Socio-demographic | Available in Private Sector Only | Health Workers | Non-availability of the vaccine in the NIP and high cost of the vaccine |  | 85.9% were aware of cervical cancer and 84.2% knew that HPV was the causative agent | 62.7% respondents were aware of the HPV vaccine and 91% of them were willing to recommend it | Reasons for non-vaccination included cost (92%) and limited access to the vaccine (76%) |
| 47 | Umeh et al. 2016 | Mothers' willingness to pay for HPV vaccines in Anambra state, Nigeria: A cross sectional contingent valuation study | Nigeria | Socio-economic | Available in Private Sector Only | Health financing | High cost of the HPV vaccine | Availability of GAVI funding and support | Demand for HPV vaccine was high. A total of 91.6% of mothers were willing to pay for the HPV vaccine | Majority of the mothers were willing to pay an average of US$ 11.68 to get their daughters fully vaccinated | At the GAVI vaccine price, up to US$ 7.58 is needed to augment the cost of the vaccine |
| 48 | Urasa and Darj 2011 | Knowledge of cervical cancer and screening practices of nurses at a regional hospital in Tanzania | Tanzania | Socio-demographic | Available in Private Sector Only | Health Workers | Inadequate knowledge on HPV vaccination. Inadequate training |  | Only 31 (22.6%) of the respondents were aware of the HPV vaccine | Of these, 7 (22.6%) knew that vaccination should be done before sexual debut | Only 2.9% and 8% had attended relevant training seminars |
| 49 | Venturas and Umeh 2017 | Health professional feedback on HPV vaccination roll-out in a developing country | Zambia | Socio-economic | Demonstration Project | Health Workers | Inadequate training of health workers Disparities in education levels of urban vs rural communities. High cost of the vaccine |  | Misconceptions and myths surrounding the vaccine translated into a fear of the HPV vaccine within the community | Health workers who were not directly involved in the pilot program were not given HPV vaccine training | Low level of education was reported as a barrier to effective vaccine communication |
| 50 | Vermandere et al. 2015 | Implementation of an HPV vaccination program in Eldoret, Kenya: Results from a qualitative assessment by key stakeholders | Kenya | Socio-cultural | Demonstration Project | Information / Community Engagement | Inadequate IEC and engagement with fathers and male teachers. Mis-conceptions about vaccines. Health facility-based vaccine delivery | Appropriate communication about cervical cancer and HPV vaccination as a preventive measure | Few fathers had heard about the past HPV vaccination program and when they had, it was mostly through their children and wives. Health care providers failed to sensitize all teachers | There was a general low level of understanding about the potential side effects of the vaccine which negatively impacted on promotional activities | Once participants were fully informed about cervical cancer and the HPV vaccine, they were accepting the vaccine |
| 51 | Vermandere et al. 2014 | Determinants of acceptance and subsequent uptake of the HPV vaccine in a cohort in Eldoret, Kenya | Kenya | Socio-demographic | Demonstration Project | Information / Community Engagement | Weak IEC strategies on the HPV vaccine |  | Up to 59.4% considered a lack of information as potentially preventing them from vaccinating their daughter. Older mothers were more likely to accept the vaccine | Baseline acceptance was associated with subsequent uptake of the vaccine. Being well-informed increased the odds of immunization | Barriers-faced leading to non-uptake: poorly informed regarding where and when immunization was to take place; fear of side effects |
| 52 | Wamai et al. 2013 | Awareness, knowledge and beliefs about HPV, cervical cancer and HPV vaccines among nurses in Cameroon: An exploratory study | Cameroon | Socio-economic | Demonstration Project | Health Workers | Low level of knowledge about the safety of the vaccine among health workers |  | 90.8% of the nursing staff surveyed acknowledged that cervical cancer is caused by HPV infection | 78.9% recognized HPV vaccine as an important means of prevention against HPV infections and 78.9% believed the vaccine is safe | 69.7% of the nurses stated that they would often recommend the HPV vaccine although, 63.9% of them said they were concerned about the side-effects |
| 53 | Watson-Jones et al. 2015 | Access and attitudes to HPV vaccination amongst hard-to-reach populations in Kenya | Kenya | Socio-economic | Demonstration Project | Service Delivery | Poor physical access to schools and health facilities. Inadequate information about HPV vaccine | Community health workers as source of information and drivers of social mobilization. Adequate and appropriate sensitization for hard-to-reach communities | Barriers to school-based delivery: long distances (up to 10km) and concerns about encountering wildlife on the way were cited as non- or delayed enrolment or absence from school | Barriers to health facility-based delivery: physical inaccessibility, complaints about the quality of care, a preference for traditional medicine | Most parents were willing for their daughters to be vaccinated after being sensitized |
| 54 | Watson-Jones et al. 2012 | Reasons for receiving or not receiving HPV vaccination in primary schoolgirls in Tanzania: A case control study | Tanzania | Socio-economic | Demonstration Project | Information / Community Engagement | Inadequate IEC about the HPV vaccine, its safety and efficacy |  | Reasons for refusing to vaccinate: absence from school, caregivers’ dissent, girls’ dissent | Vaccine acceptance was associated with an understanding of the benefits of the vaccine in preventing cervical cancer | Fear of side effects, infertility and insufficient knowledge about the vaccine was associated with vaccine refusal |
